# Supplementary material for: Mast4 determines the cell fate of MSCs for bone and cartilage development
Source: Nat Commun. 2022 Jul 8;13:3960. doi: 10.1038/s41467-022-31697-3 (PMC9270402; doi:10.1038/s41467-022-31697-3)
Supplement: Supplementary file 1 — Supplementary Information [file 41467_2022_31697_MOESM1_ESM.pptx]

## Slide 1
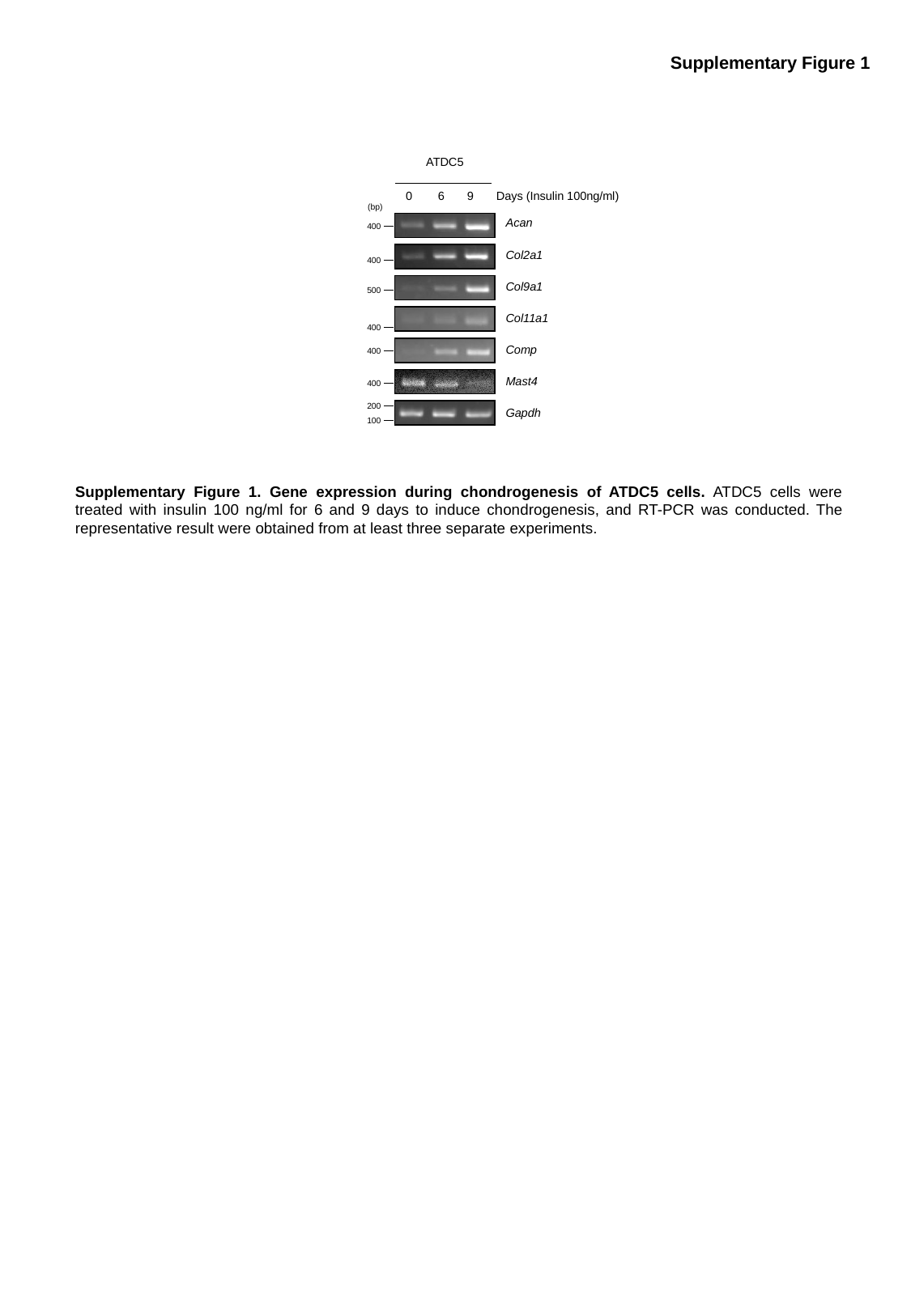

Supplementary Figure 1
ATDC5
0 6 9 Days (Insulin 100ng/ml)
(bp)
Acan
400
Col2a1
400
Col9a1
500
Col11a1
400
Comp
400
Mast4
400
200
Gapdh
100
Supplementary Figure 1. Gene expression during chondrogenesis of ATDC5 cells. ATDC5 cells were treated with insulin 100 ng/ml for 6 and 9 days to induce chondrogenesis, and RT-PCR was conducted. The representative result were obtained from at least three separate experiments.

## Slide 2
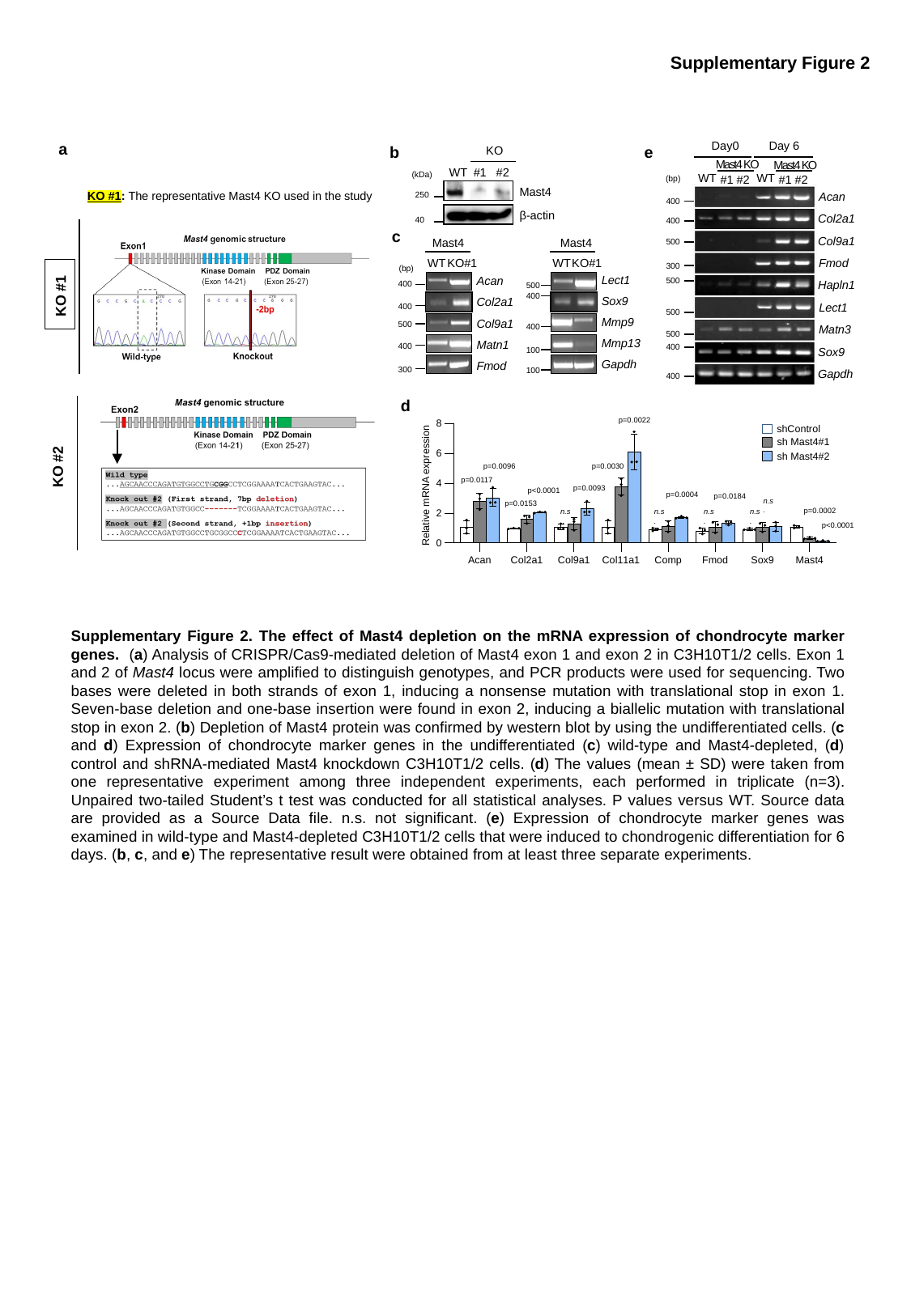

Supplementary Figure 2
Day0
Day 6
a
b
e
 KO
WT #1 #2
Mast4
β-actin
Mast4 KO
Mast4 KO
(kDa)
WT
WT
#1 #2
#1 #2
(bp)
KO #1: The representative Mast4 KO used in the study
Acan
250
400
Col2a1
40
400
c
Col9a1
Mast4
Mast4
500
Fmod
WT
KO#1
WT
 KO#1
300
(bp)
Lect1
Acan
500
Hapln1
400
500
KO #1
400
Sox9
Col2a1
Lect1
400
500
Mmp9
Col9a1
500
400
Matn3
500
Mmp13
Matn1
400
400
Sox9
100
Gapdh
Fmod
300
100
Gapdh
400
d
p=0.0022
shControl
sh Mast4#1
sh Mast4#2
KO #2
p=0.0096
p=0.0030
p=0.0117
Relative mRNA expression
p=0.0093
p<0.0001
p=0.0004
p=0.0184
n.s.
p=0.0153
p=0.0002
n.s..
n.s.
n.s.
n.s.
p<0.0001
Supplementary Figure 2. The effect of Mast4 depletion on the mRNA expression of chondrocyte marker genes. (a) Analysis of CRISPR/Cas9-mediated deletion of Mast4 exon 1 and exon 2 in C3H10T1/2 cells. Exon 1 and 2 of Mast4 locus were amplified to distinguish genotypes, and PCR products were used for sequencing. Two bases were deleted in both strands of exon 1, inducing a nonsense mutation with translational stop in exon 1. Seven-base deletion and one-base insertion were found in exon 2, inducing a biallelic mutation with translational stop in exon 2. (b) Depletion of Mast4 protein was confirmed by western blot by using the undifferentiated cells. (c and d) Expression of chondrocyte marker genes in the undifferentiated (c) wild-type and Mast4-depleted, (d) control and shRNA-mediated Mast4 knockdown C3H10T1/2 cells. (d) The values (mean ± SD) were taken from one representative experiment among three independent experiments, each performed in triplicate (n=3). Unpaired two-tailed Student’s t test was conducted for all statistical analyses. P values versus WT. Source data are provided as a Source Data file. n.s. not significant. (e) Expression of chondrocyte marker genes was examined in wild-type and Mast4-depleted C3H10T1/2 cells that were induced to chondrogenic differentiation for 6 days. (b, c, and e) The representative result were obtained from at least three separate experiments.

## Slide 3
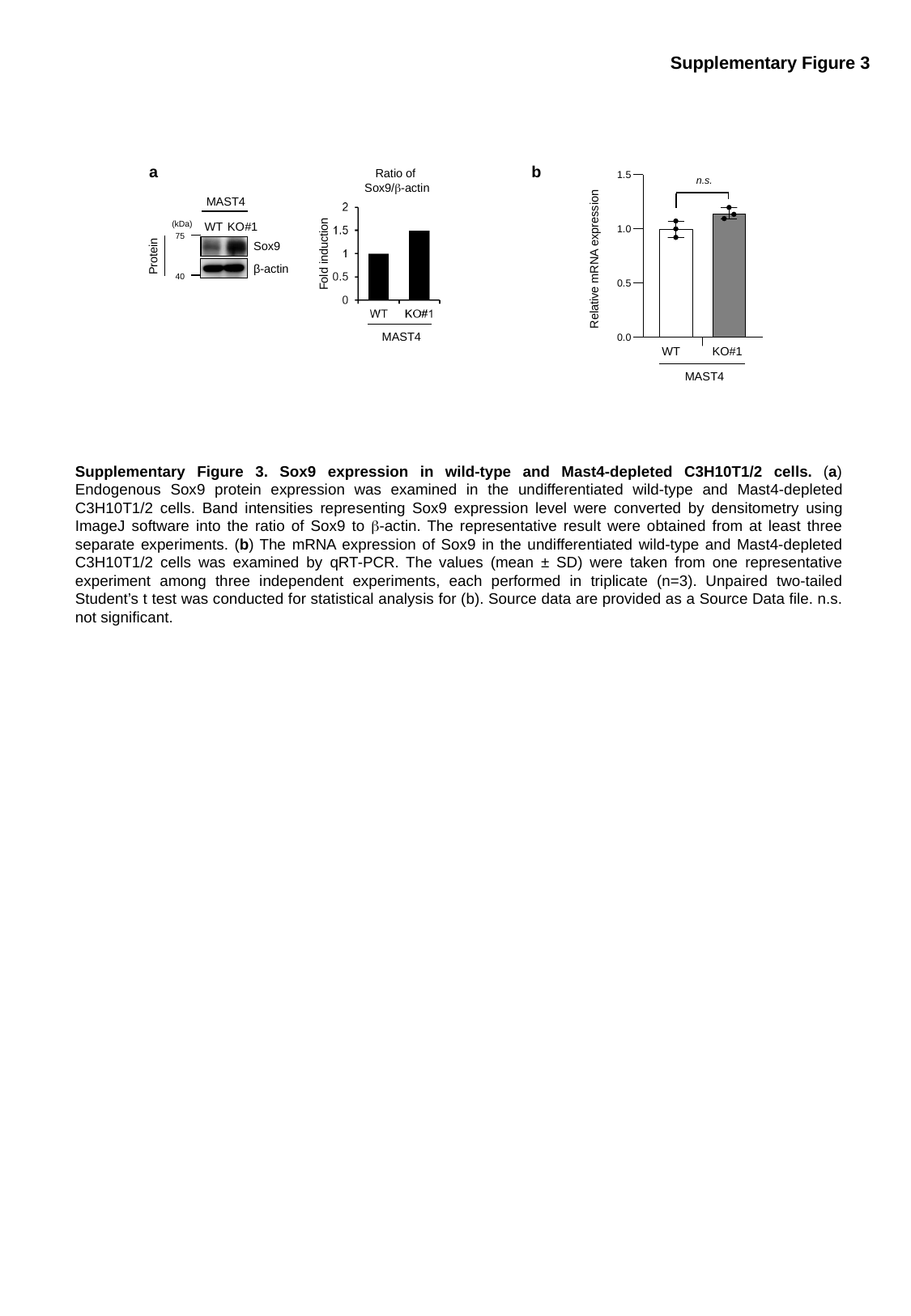

Supplementary Figure 3
a
b
Ratio of
Sox9/b-actin
n.s.
MAST4
(kDa)
#1
WT
KO
Fold induction
75
Sox9
Relative mRNA expression
Protein
β-actin
40
MAST4
WT
KO#1
MAST4
Supplementary Figure 3. Sox9 expression in wild-type and Mast4-depleted C3H10T1/2 cells. (a) Endogenous Sox9 protein expression was examined in the undifferentiated wild-type and Mast4-depleted C3H10T1/2 cells. Band intensities representing Sox9 expression level were converted by densitometry using ImageJ software into the ratio of Sox9 to b-actin. The representative result were obtained from at least three separate experiments. (b) The mRNA expression of Sox9 in the undifferentiated wild-type and Mast4-depleted C3H10T1/2 cells was examined by qRT-PCR. The values (mean ± SD) were taken from one representative experiment among three independent experiments, each performed in triplicate (n=3). Unpaired two-tailed Student’s t test was conducted for statistical analysis for (b). Source data are provided as a Source Data file. n.s. not significant.

## Slide 4
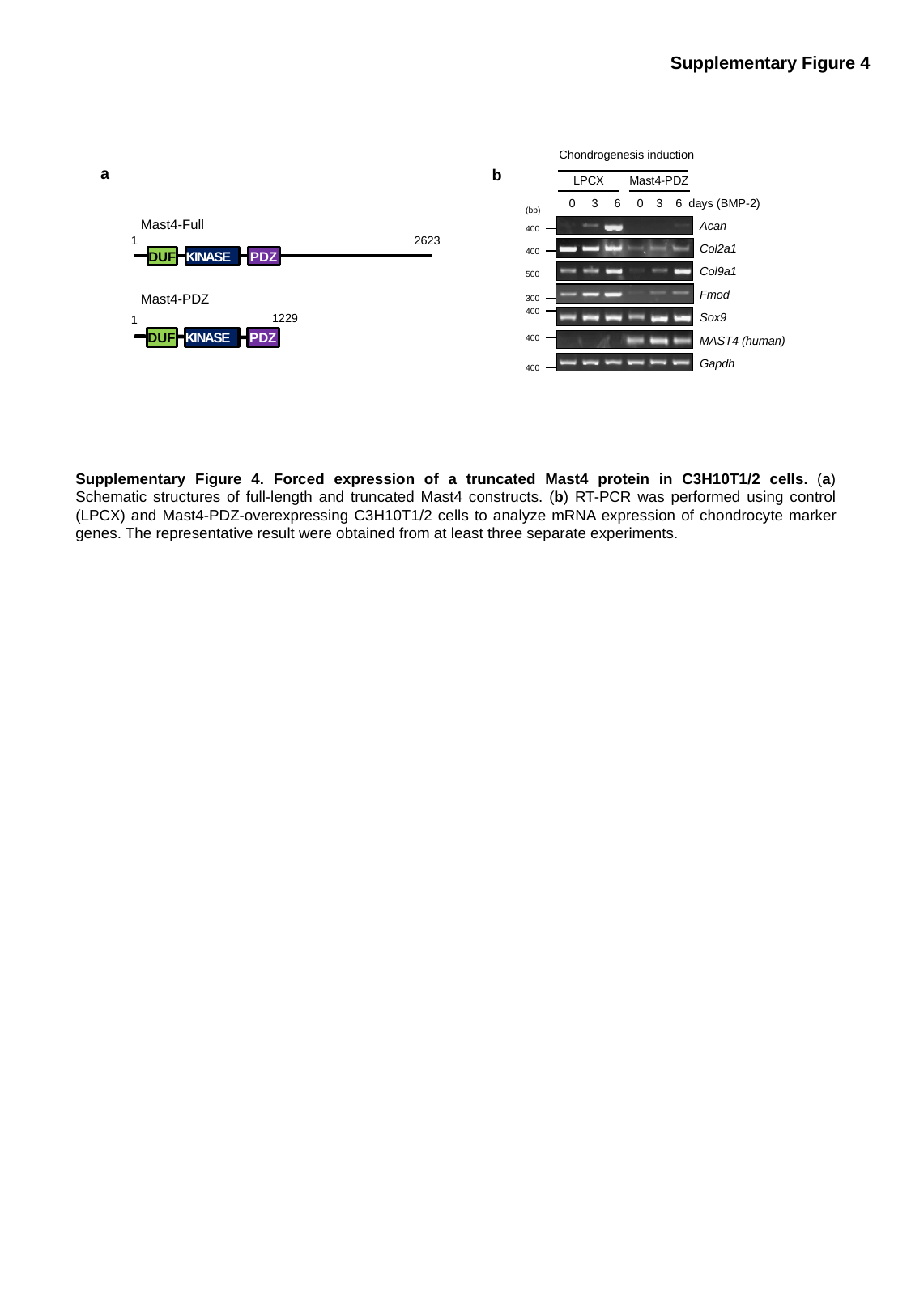

Supplementary Figure 4
Chondrogenesis induction
a
b
LPCX
Mast4-PDZ
0 3 6 0 3 6 days (BMP-2)
(bp)
Mast4-Full
Acan
400
1
2623
Col2a1
400
KINASE
PDZ
DUF
Col9a1
500
Fmod
Mast4-PDZ
300
400
Sox9
1229
1
400
MAST4 (human)
DUF
KINASE
PDZ
Gapdh
400
Supplementary Figure 4. Forced expression of a truncated Mast4 protein in C3H10T1/2 cells. (a) Schematic structures of full-length and truncated Mast4 constructs. (b) RT-PCR was performed using control (LPCX) and Mast4-PDZ-overexpressing C3H10T1/2 cells to analyze mRNA expression of chondrocyte marker genes. The representative result were obtained from at least three separate experiments.

## Slide 5
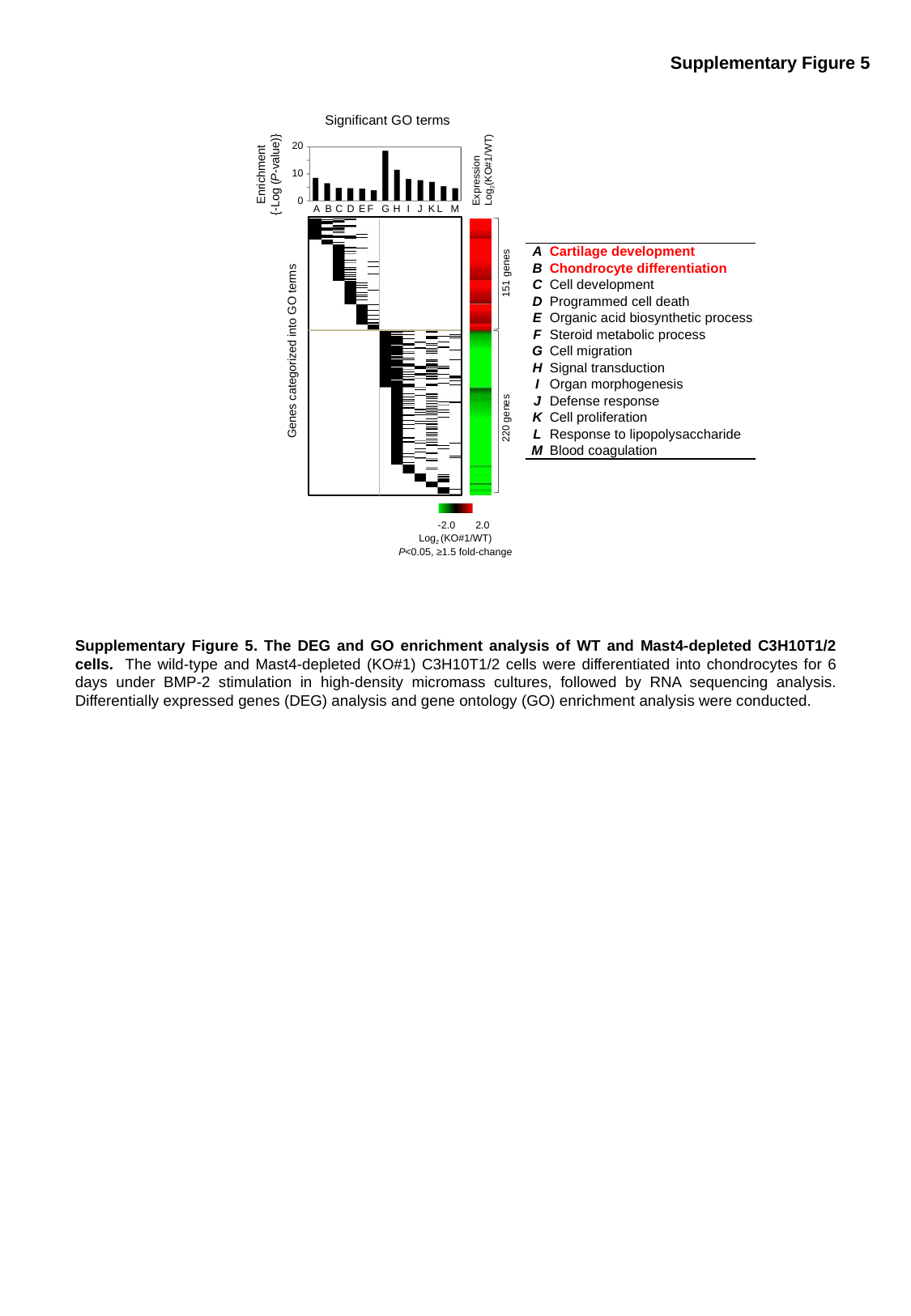

Supplementary Figure 5
Significant GO terms
Expression
Log2(KO#1/WT)
151 genes
220 genes
20
Enrichment
{-Log (P-value)}
10
0
A
B
C
D
E
F
G
H
I
J
L
M
K
Genes categorized into GO terms
| A | Cartilage development |
| --- | --- |
| B | Chondrocyte differentiation |
| C | Cell development |
| D | Programmed cell death |
| E | Organic acid biosynthetic process |
| F | Steroid metabolic process |
| G | Cell migration |
| H | Signal transduction |
| I | Organ morphogenesis |
| J | Defense response |
| K | Cell proliferation |
| L | Response to lipopolysaccharide |
| M | Blood coagulation |
-2.0
2.0
Log2 (KO#1/WT)
P<0.05, ≥1.5 fold-change
Supplementary Figure 5. The DEG and GO enrichment analysis of WT and Mast4-depleted C3H10T1/2 cells. The wild-type and Mast4-depleted (KO#1) C3H10T1/2 cells were differentiated into chondrocytes for 6 days under BMP-2 stimulation in high-density micromass cultures, followed by RNA sequencing analysis. Differentially expressed genes (DEG) analysis and gene ontology (GO) enrichment analysis were conducted.

## Slide 6
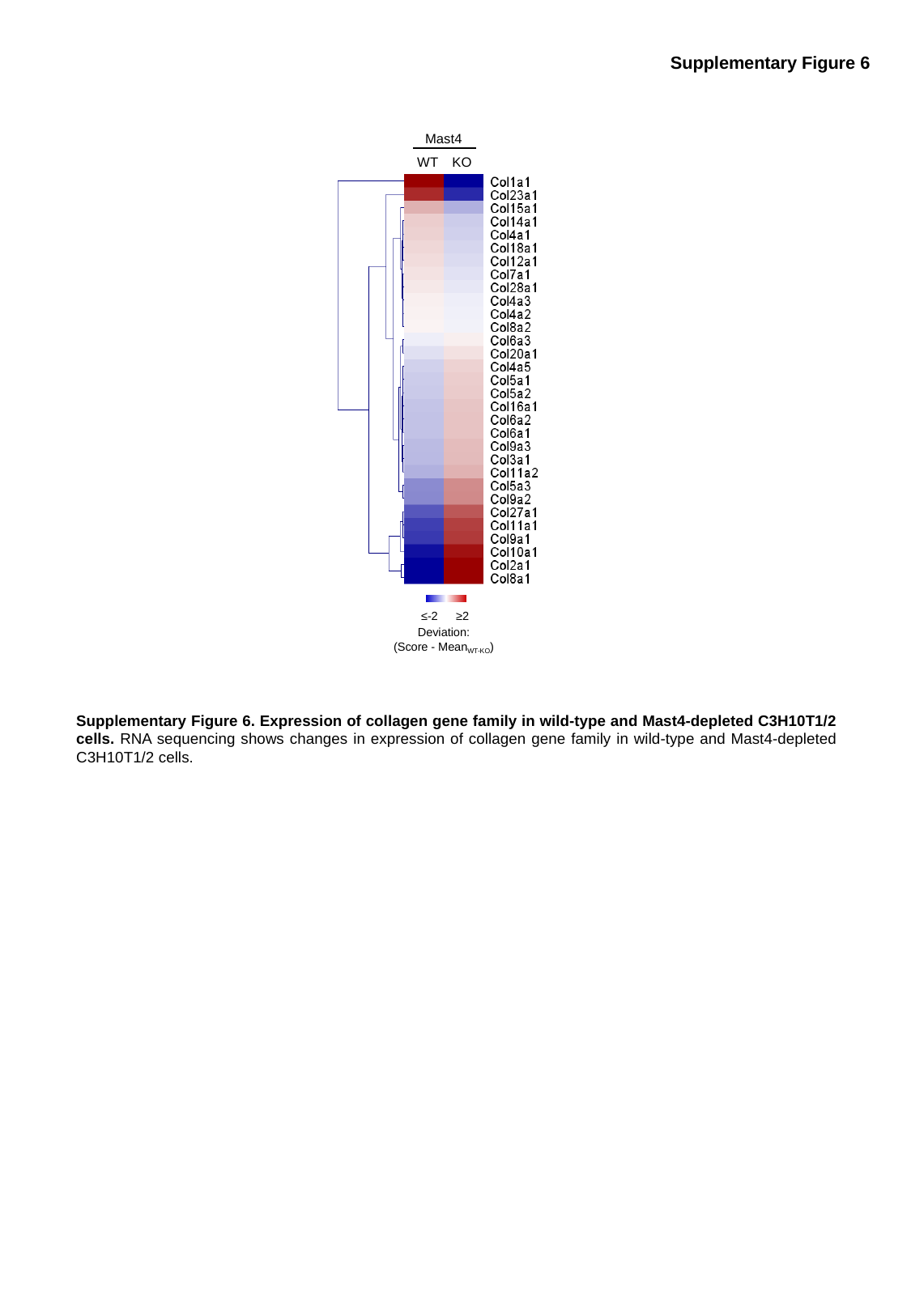

Supplementary Figure 6
Mast4
WT
KO
≤-2
≥2
Deviation:
(Score - MeanWT-KO)
Supplementary Figure 6. Expression of collagen gene family in wild-type and Mast4-depleted C3H10T1/2 cells. RNA sequencing shows changes in expression of collagen gene family in wild-type and Mast4-depleted C3H10T1/2 cells.

## Slide 7
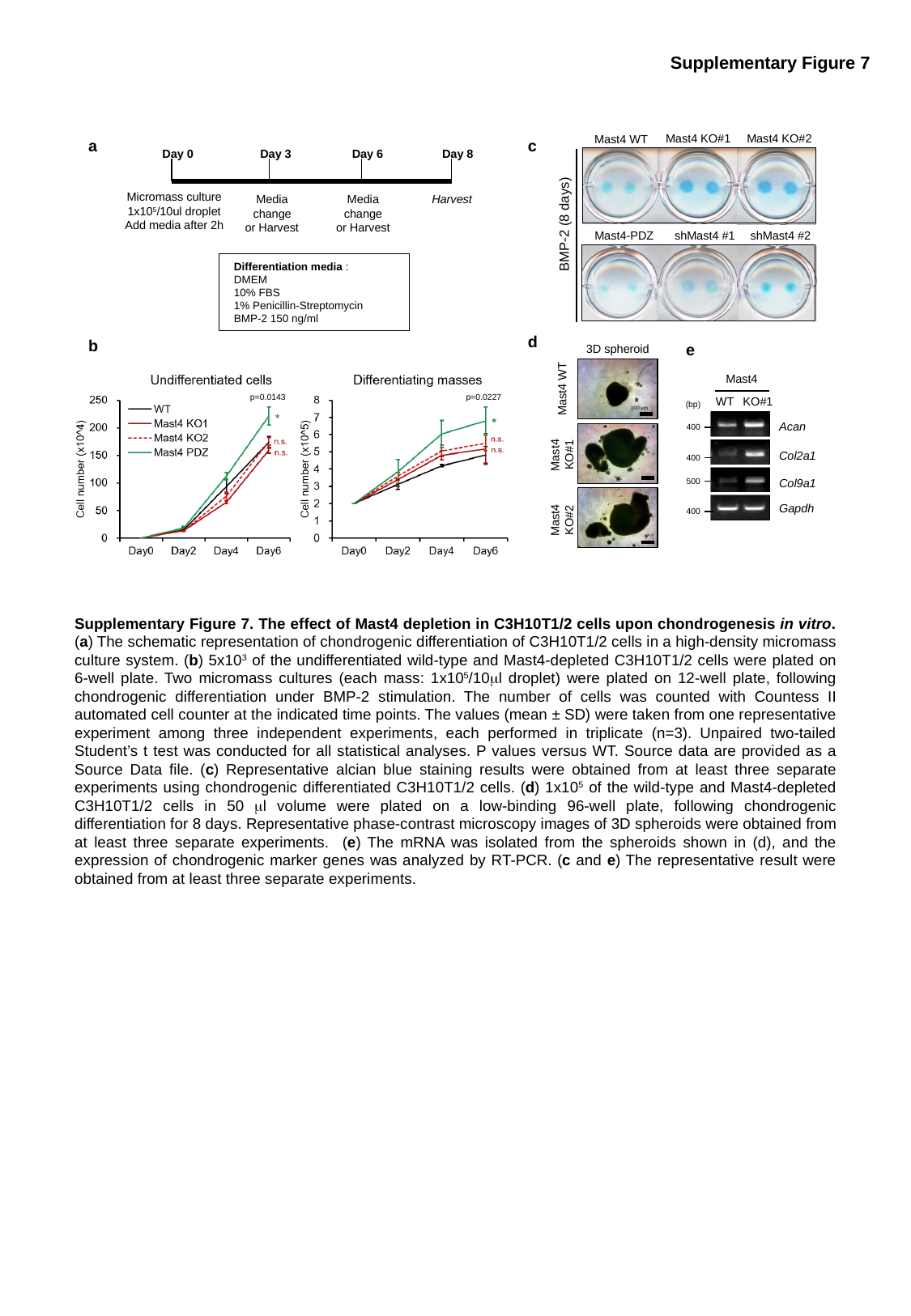

Supplementary Figure 7
Mast4 KO#1
Mast4 KO#2
Mast4 WT
Mast4-PDZ
shMast4 #1
shMast4 #2
BMP-2 (8 days)
a
c
Day 0
Day 3
Day 6
Day 8
Micromass culture
1x105/10ul droplet
Add media after 2h
Media change
or Harvest
Media change
or Harvest
Harvest
 Differentiation media :
 DMEM
 10% FBS
 1% Penicillin-Streptomycin
 BMP-2 150 ng/ml
d
b
e
3D spheroid
Mast4 WT
100 mm
Mast4 KO#1
Mast4 KO#2
Mast4
 p=0.0227
 p=0.0143
 KO#1
WT
(bp)
Acan
400
Col2a1
400
Col9a1
500
Gapdh
400
Supplementary Figure 7. The effect of Mast4 depletion in C3H10T1/2 cells upon chondrogenesis in vitro. (a) The schematic representation of chondrogenic differentiation of C3H10T1/2 cells in a high-density micromass culture system. (b) 5x10^3^ of the undifferentiated wild-type and Mast4-depleted C3H10T1/2 cells were plated on 6-well plate. Two micromass cultures (each mass: 1x105/10ml droplet) were plated on 12-well plate, following chondrogenic differentiation under BMP-2 stimulation. The number of cells was counted with Countess II automated cell counter at the indicated time points. The values (mean ± SD) were taken from one representative experiment among three independent experiments, each performed in triplicate (n=3). Unpaired two-tailed Student’s t test was conducted for all statistical analyses. P values versus WT. Source data are provided as a Source Data file. (c) Representative alcian blue staining results were obtained from at least three separate experiments using chondrogenic differentiated C3H10T1/2 cells. (d) 1x10^5^ of the wild-type and Mast4-depleted C3H10T1/2 cells in 50 ml volume were plated on a low-binding 96-well plate, following chondrogenic differentiation for 8 days. Representative phase-contrast microscopy images of 3D spheroids were obtained from at least three separate experiments. (e) The mRNA was isolated from the spheroids shown in (d), and the expression of chondrogenic marker genes was analyzed by RT-PCR. (c and e) The representative result were obtained from at least three separate experiments.

## Slide 8
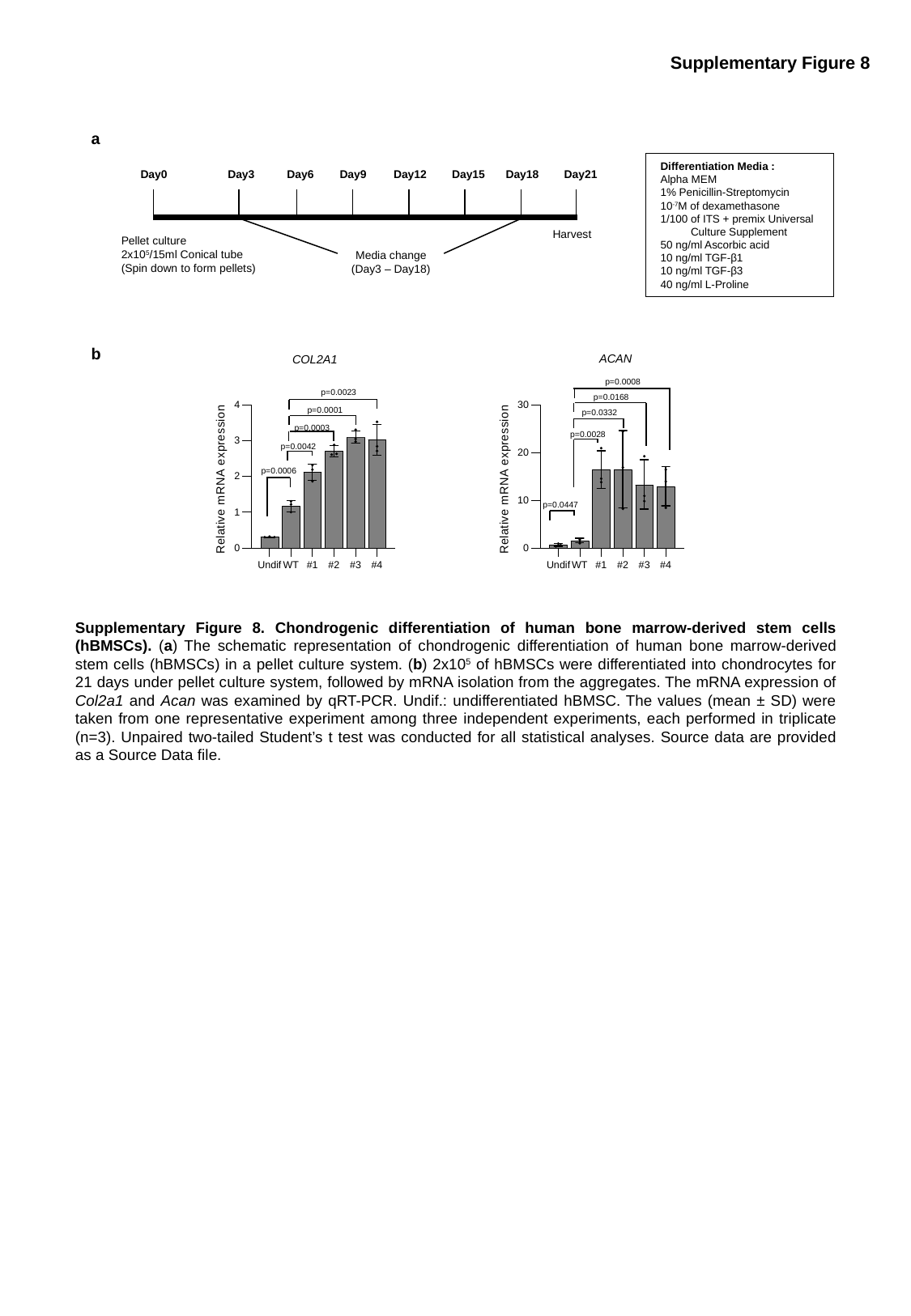

Supplementary Figure 8
a
 Differentiation Media :
 Alpha MEM
 1% Penicillin-Streptomycin
 10-7M of dexamethasone
 1/100 of ITS + premix Universal
 Culture Supplement
 50 ng/ml Ascorbic acid
 10 ng/ml TGF-β1
 10 ng/ml TGF-β3
 40 ng/ml L-Proline
Day0
Day3
Day9
Day12
Day15
Day18
Day21
Day6
Harvest
Pellet culture
2x105/15ml Conical tube
(Spin down to form pellets)
Media change
(Day3 – Day18)
b
ACAN
COL2A1
 p=0.0008
 p=0.0168
 p=0.0332
 p=0.0028
 p=0.0447
 p=0.0023
 p=0.0001
 p=0.0003
 p=0.0042
 p=0.0006
Supplementary Figure 8. Chondrogenic differentiation of human bone marrow-derived stem cells (hBMSCs). (a) The schematic representation of chondrogenic differentiation of human bone marrow-derived stem cells (hBMSCs) in a pellet culture system. (b) 2x10^5^ of hBMSCs were differentiated into chondrocytes for 21 days under pellet culture system, followed by mRNA isolation from the aggregates. The mRNA expression of Col2a1 and Acan was examined by qRT-PCR. Undif.: undifferentiated hBMSC. The values (mean ± SD) were taken from one representative experiment among three independent experiments, each performed in triplicate (n=3). Unpaired two-tailed Student’s t test was conducted for all statistical analyses. Source data are provided as a Source Data file.

## Slide 9
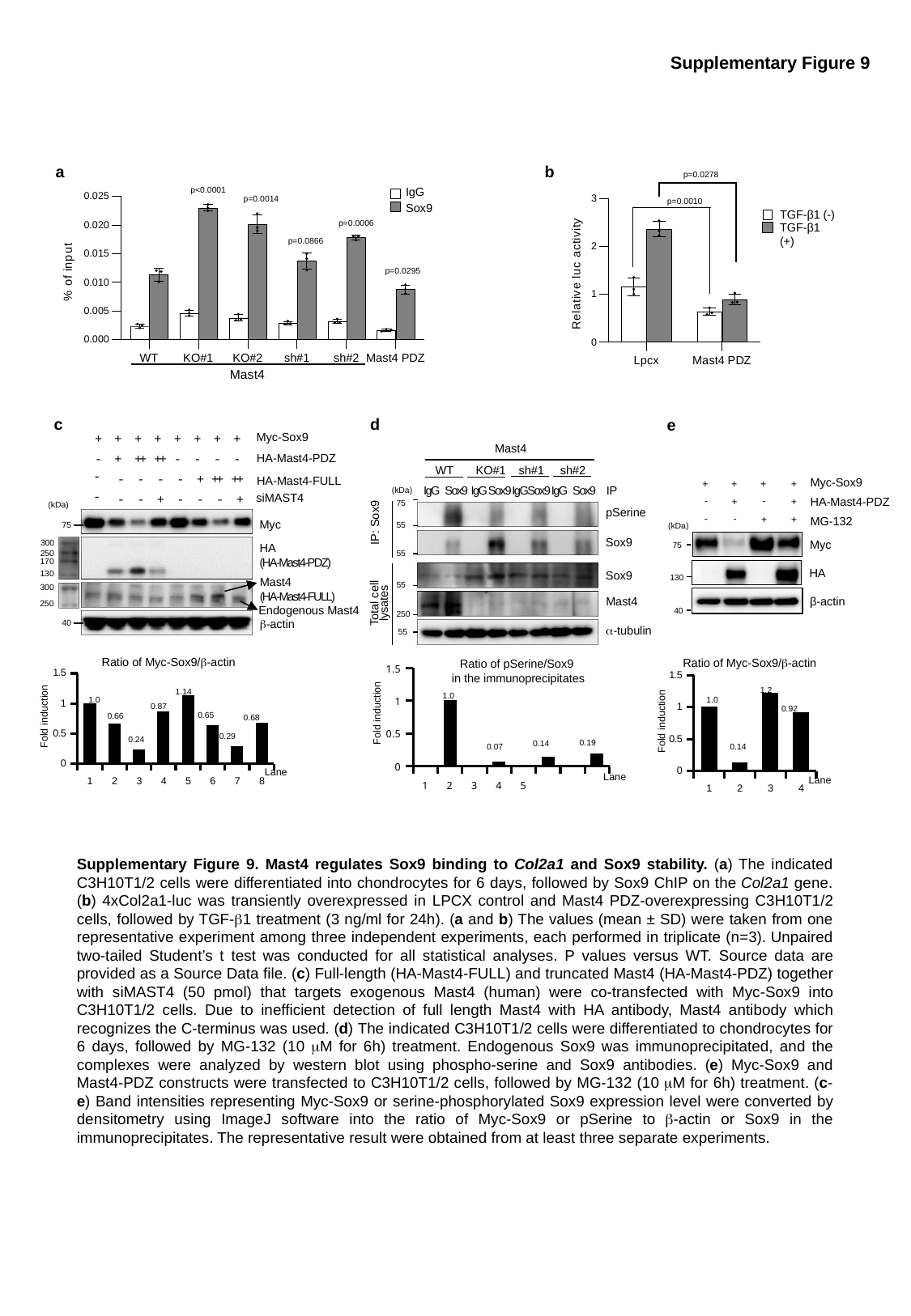

Supplementary Figure 9
a
b
p=0.0278
IgG
p<0.0001
p=0.0014
p=0.0010
Sox9
TGF-β1 (-)
p=0.0006
TGF-β1 (+)
p=0.0866
p=0.0295
Mast4
c
d
e
+ + + + + + + +
- + ++ ++ - - - -
- - - - + ++ ++
- - + - - - +
Myc-Sox9
Mast4
WT KO#1 sh#1 sh#2
IgG Sox9 IgG Sox9 IgGSox9 IgG Sox9 IP
(kDa)
75
pSerine
IP: Sox9
55
Sox9
55
Sox9
55
Mast4
250
a-tubulin
55
HA-Mast4-PDZ
HA-Mast4-FULL
Myc-Sox9
+
+
+
+
siMAST4
-
-
HA-Mast4-PDZ
+
+
(kDa)
-
-
+
+
MG-132
Myc
75
(kDa)
300
Myc
75
HA
(HA-Mast4-PDZ)
250
Total cell lysates
170
HA
130
130
Mast4
(HA-Mast4-FULL)
300
β-actin
250
Endogenous Mast4
40
b-actin
40
Ratio of Myc-Sox9/b-actin
### Chart
| Category | |
|---|---|Fold induction
1.14
1.0
0.87
0.65
0.66
0.68
0.29
0.24
Lane
Ratio of Myc-Sox9/b-actin
### Chart
| Category | |
|---|---|Fold induction
1.2
1.0
0.92
0.14
Lane
Ratio of pSerine/Sox9
in the immunoprecipitates
### Chart
| Category | |
|---|---|Fold induction
1.0
0.19
0.14
0.07
Lane
Supplementary Figure 9. Mast4 regulates Sox9 binding to Col2a1 and Sox9 stability. (a) The indicated C3H10T1/2 cells were differentiated into chondrocytes for 6 days, followed by Sox9 ChIP on the Col2a1 gene. (b) 4xCol2a1-luc was transiently overexpressed in LPCX control and Mast4 PDZ-overexpressing C3H10T1/2 cells, followed by TGF-b1 treatment (3 ng/ml for 24h). (a and b) The values (mean ± SD) were taken from one representative experiment among three independent experiments, each performed in triplicate (n=3). Unpaired two-tailed Student’s t test was conducted for all statistical analyses. P values versus WT. Source data are provided as a Source Data file. (c) Full-length (HA-Mast4-FULL) and truncated Mast4 (HA-Mast4-PDZ) together with siMAST4 (50 pmol) that targets exogenous Mast4 (human) were co-transfected with Myc-Sox9 into C3H10T1/2 cells. Due to inefficient detection of full length Mast4 with HA antibody, Mast4 antibody which recognizes the C-terminus was used. (d) The indicated C3H10T1/2 cells were differentiated to chondrocytes for 6 days, followed by MG-132 (10 mM for 6h) treatment. Endogenous Sox9 was immunoprecipitated, and the complexes were analyzed by western blot using phospho-serine and Sox9 antibodies. (e) Myc-Sox9 and Mast4-PDZ constructs were transfected to C3H10T1/2 cells, followed by MG-132 (10 mM for 6h) treatment. (c-e) Band intensities representing Myc-Sox9 or serine-phosphorylated Sox9 expression level were converted by densitometry using ImageJ software into the ratio of Myc-Sox9 or pSerine to b-actin or Sox9 in the immunoprecipitates. The representative result were obtained from at least three separate experiments.

## Slide 10
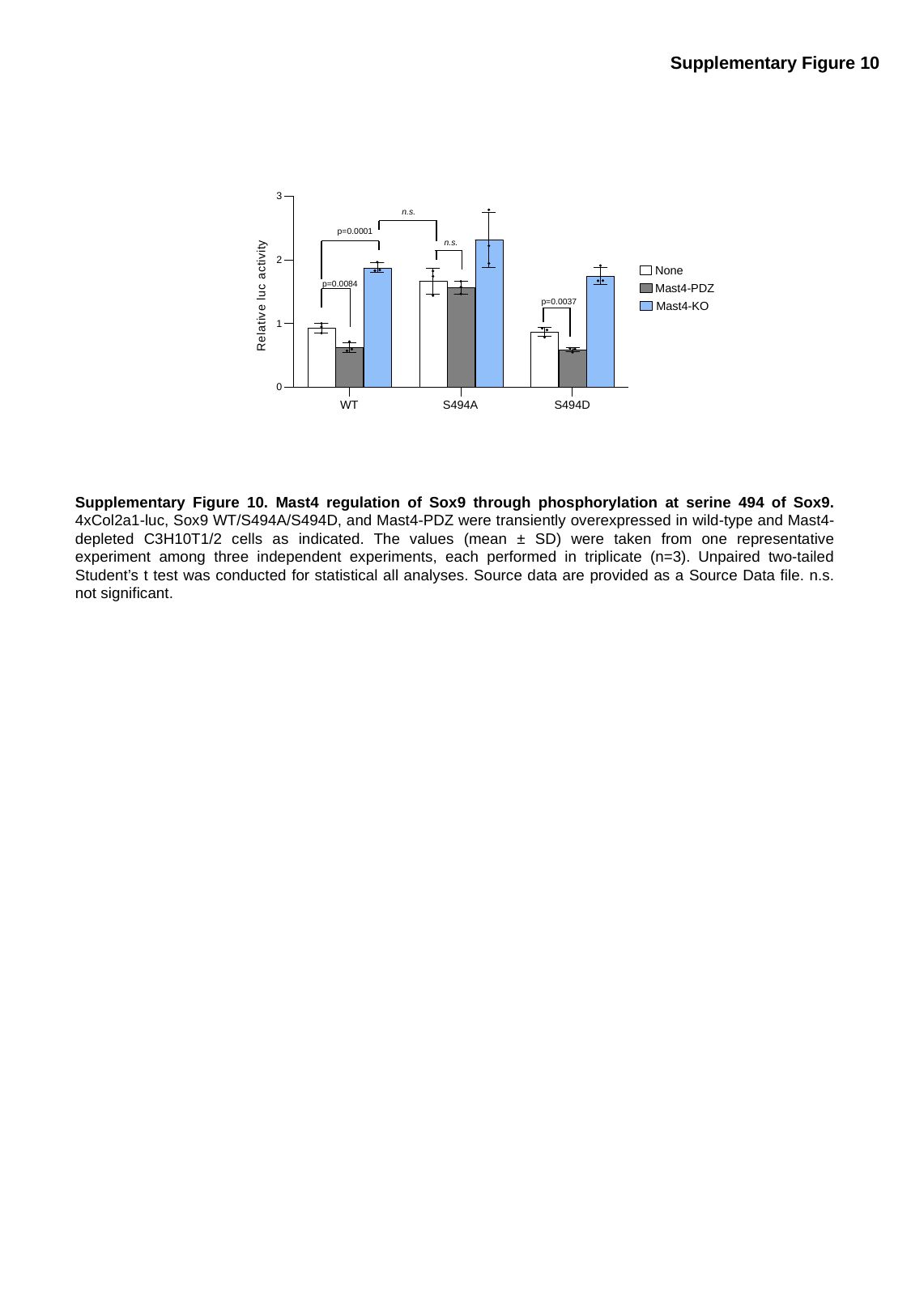

Supplementary Figure 10
n.s.
p=0.0001
n.s.
None
p=0.0084
Mast4-PDZ
p=0.0037
Mast4-KO
Supplementary Figure 10. Mast4 regulation of Sox9 through phosphorylation at serine 494 of Sox9. 4xCol2a1-luc, Sox9 WT/S494A/S494D, and Mast4-PDZ were transiently overexpressed in wild-type and Mast4-depleted C3H10T1/2 cells as indicated. The values (mean ± SD) were taken from one representative experiment among three independent experiments, each performed in triplicate (n=3). Unpaired two-tailed Student’s t test was conducted for statistical all analyses. Source data are provided as a Source Data file. n.s. not significant.

## Slide 11
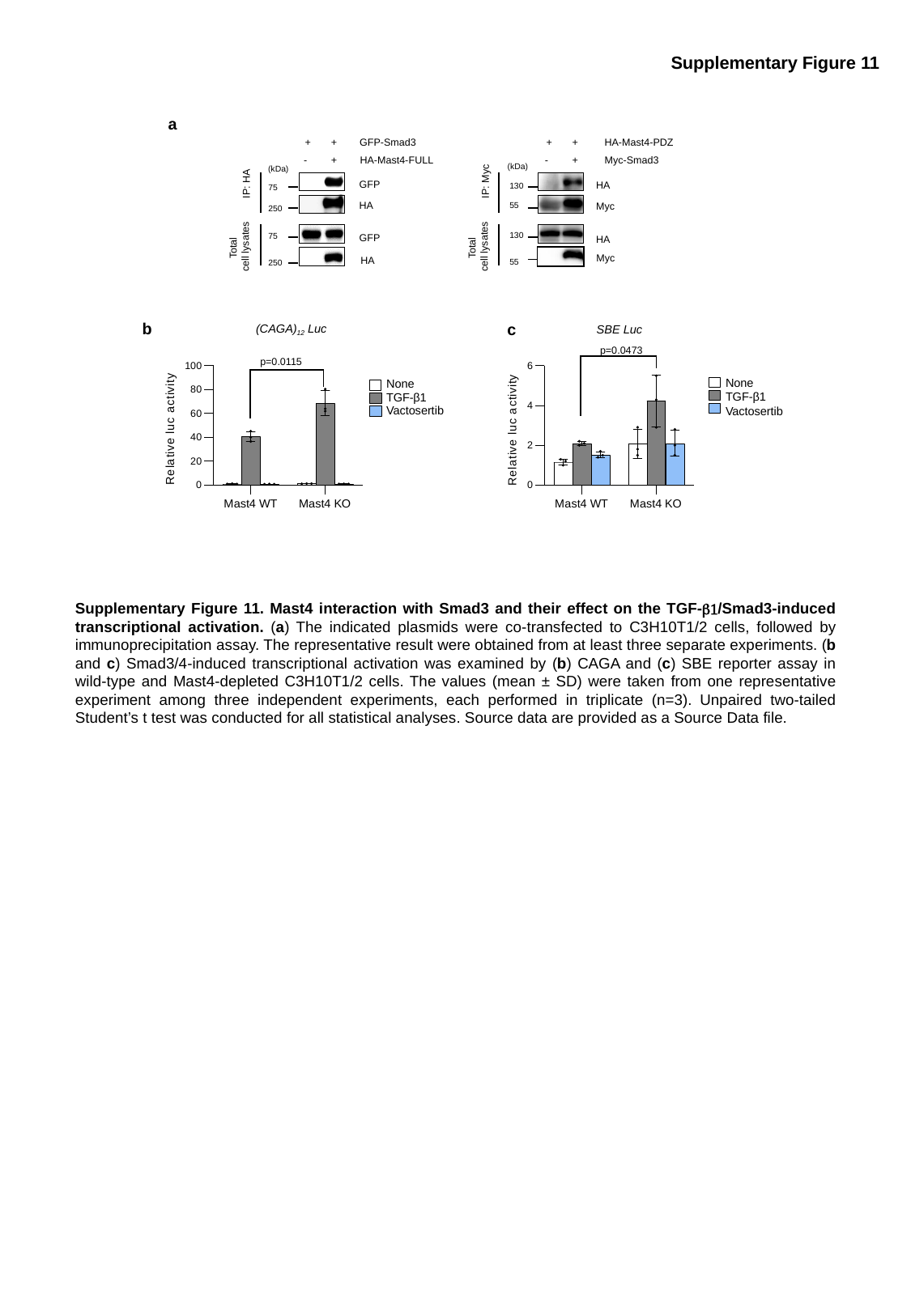

Supplementary Figure 11
a
GFP-Smad3
HA-Mast4-PDZ
+
+
+
+
-
+
HA-Mast4-FULL
-
+
Myc-Smad3
(kDa)
(kDa)
IP: Myc
IP: HA
GFP
HA
130
75
HA
55
Myc
250
Total
cell lysates
Total
cell lysates
130
75
GFP
HA
Myc
HA
55
250
b
c
(CAGA)12 Luc
SBE Luc
p=0.0473
p=0.0115
None
None
TGF-β1
TGF-β1
Vactosertib
Vactosertib
Supplementary Figure 11. Mast4 interaction with Smad3 and their effect on the TGF-b1/Smad3-induced transcriptional activation. (a) The indicated plasmids were co-transfected to C3H10T1/2 cells, followed by immunoprecipitation assay. The representative result were obtained from at least three separate experiments. (b and c) Smad3/4-induced transcriptional activation was examined by (b) CAGA and (c) SBE reporter assay in wild-type and Mast4-depleted C3H10T1/2 cells. The values (mean ± SD) were taken from one representative experiment among three independent experiments, each performed in triplicate (n=3). Unpaired two-tailed Student’s t test was conducted for all statistical analyses. Source data are provided as a Source Data file.

## Slide 12
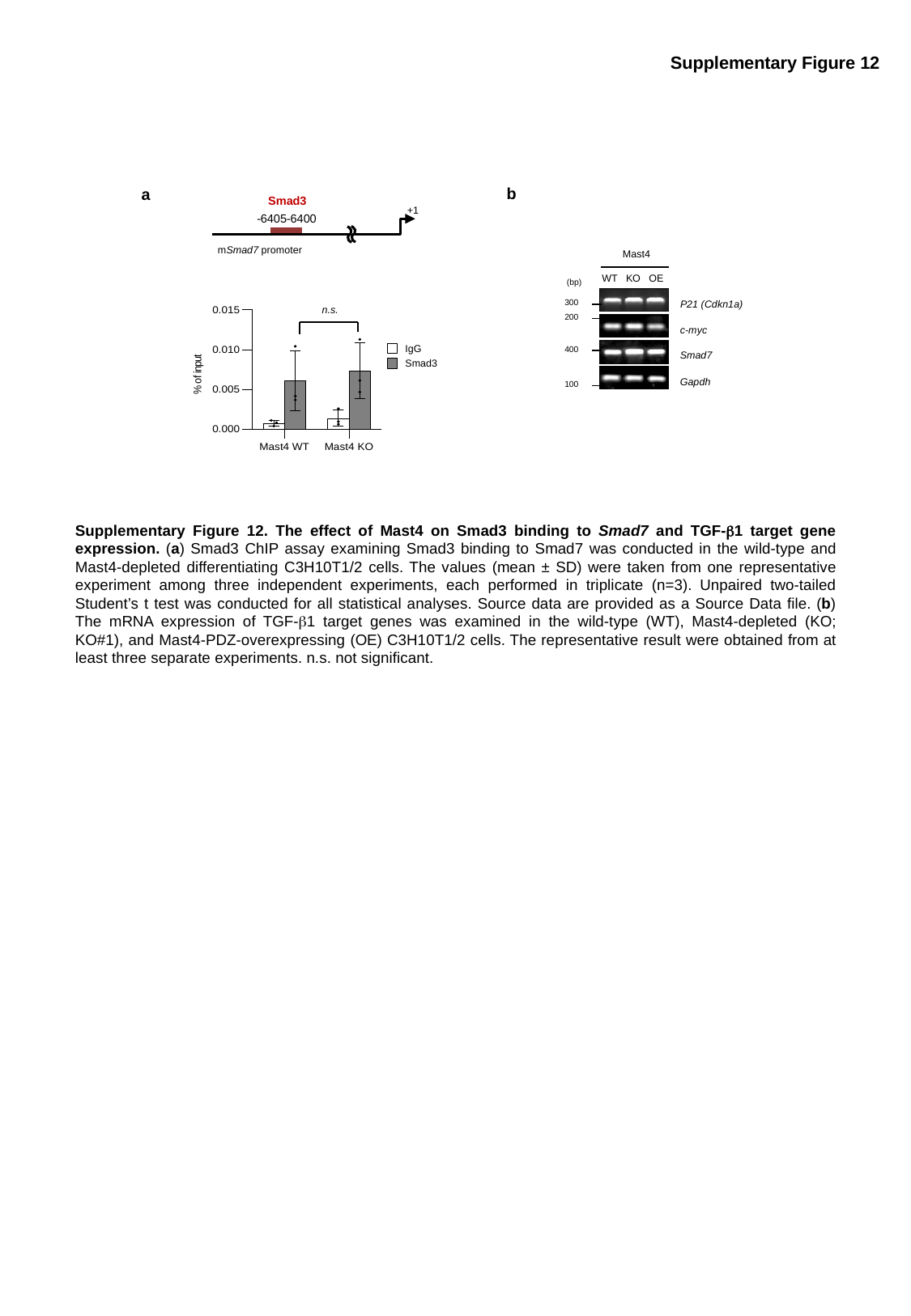

Supplementary Figure 12
b
a
Smad3
+1
-6405-6400
mSmad7 promoter
Mast4
 WT KO OE
P21 (Cdkn1a)
c-myc
Smad7
Gapdh
(bp)
300
n.s.
200
IgG
400
Smad3
100
Supplementary Figure 12. The effect of Mast4 on Smad3 binding to Smad7 and TGF-b1 target gene expression. (a) Smad3 ChIP assay examining Smad3 binding to Smad7 was conducted in the wild-type and Mast4-depleted differentiating C3H10T1/2 cells. The values (mean ± SD) were taken from one representative experiment among three independent experiments, each performed in triplicate (n=3). Unpaired two-tailed Student’s t test was conducted for all statistical analyses. Source data are provided as a Source Data file. (b) The mRNA expression of TGF-b1 target genes was examined in the wild-type (WT), Mast4-depleted (KO; KO#1), and Mast4-PDZ-overexpressing (OE) C3H10T1/2 cells. The representative result were obtained from at least three separate experiments. n.s. not significant.

## Slide 13
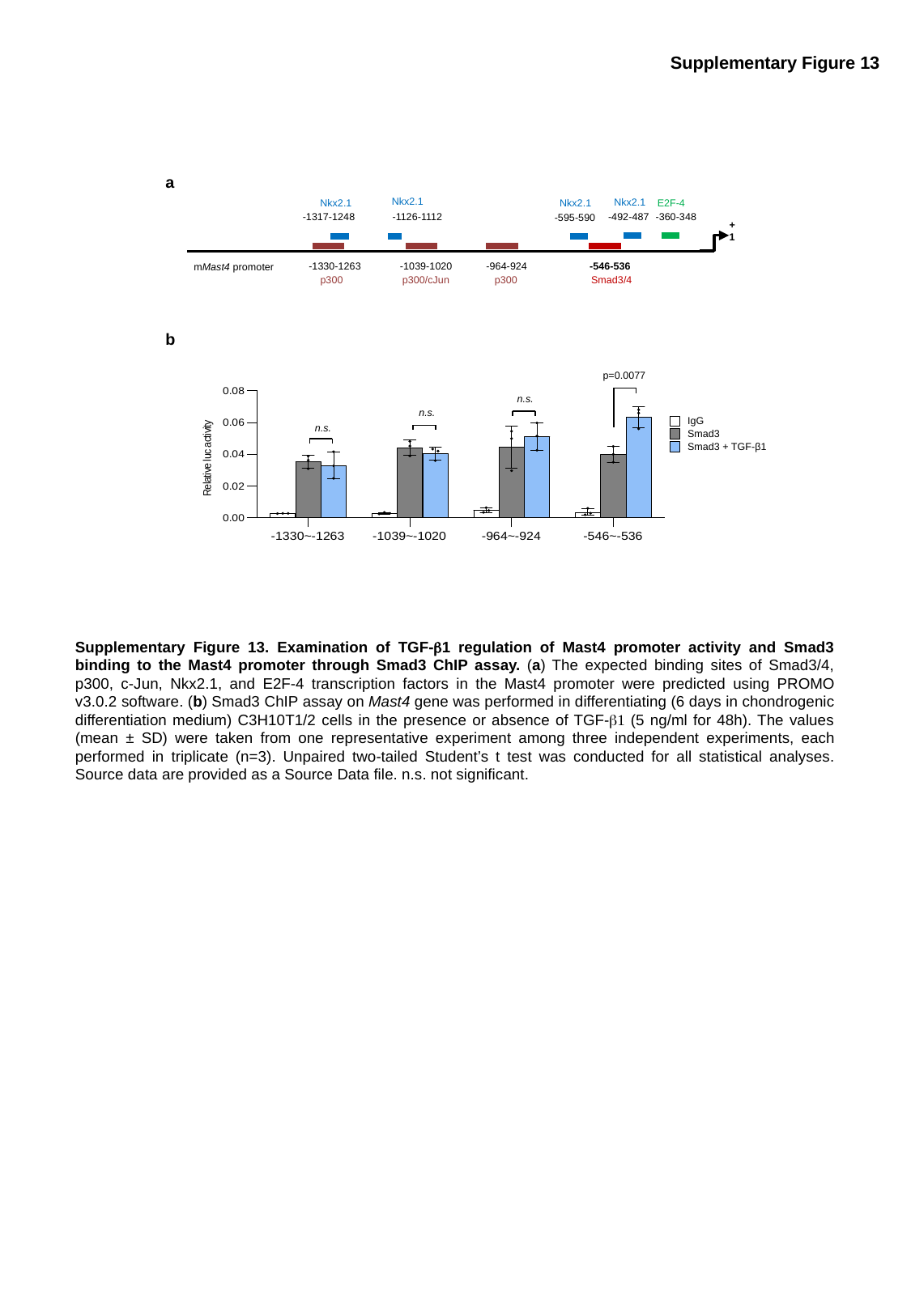

Supplementary Figure 13
a
Nkx2.1
Nkx2.1
E2F-4
Nkx2.1
Nkx2.1
-360-348
-1126-1112
-492-487
-1317-1248
-595-590
+1
-1330-1263
-964-924
-1039-1020
-546-536
mMast4 promoter
p300 p300/cJun p300 Smad3/4
b
p=0.0077
n.s.
n.s.
IgG
n.s.
Smad3
Smad3 + TGF-β1
Supplementary Figure 13. Examination of TGF-b1 regulation of Mast4 promoter activity and Smad3 binding to the Mast4 promoter through Smad3 ChIP assay. (a) The expected binding sites of Smad3/4, p300, c-Jun, Nkx2.1, and E2F-4 transcription factors in the Mast4 promoter were predicted using PROMO v3.0.2 software. (b) Smad3 ChIP assay on Mast4 gene was performed in differentiating (6 days in chondrogenic differentiation medium) C3H10T1/2 cells in the presence or absence of TGF-b1 (5 ng/ml for 48h). The values (mean ± SD) were taken from one representative experiment among three independent experiments, each performed in triplicate (n=3). Unpaired two-tailed Student’s t test was conducted for all statistical analyses. Source data are provided as a Source Data file. n.s. not significant.

## Slide 14
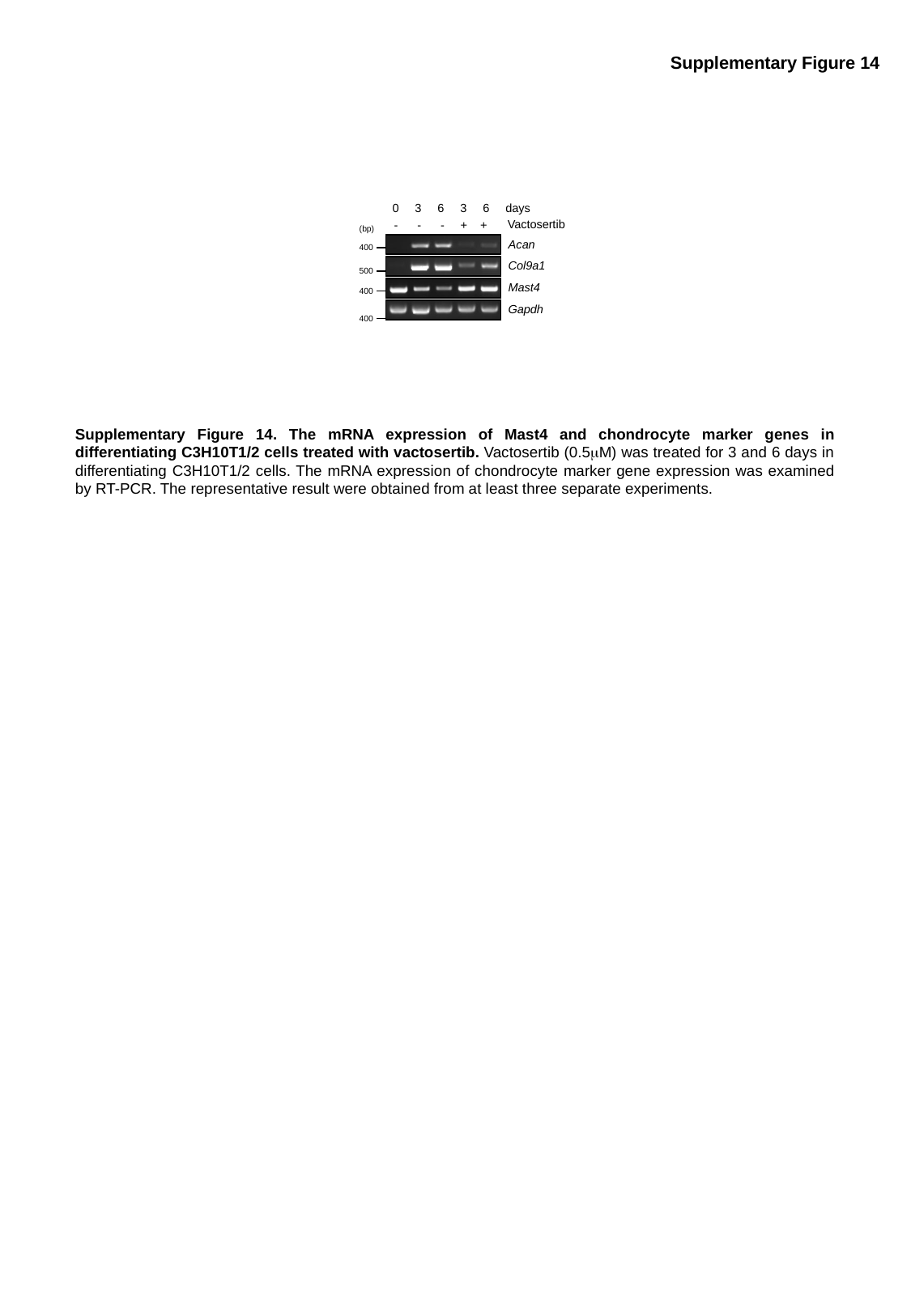

Supplementary Figure 14
 0 3 6 3 6 days
Vactosertib
 - - - + +
(bp)
Acan
400
Col9a1
500
Mast4
400
Gapdh
400
Supplementary Figure 14. The mRNA expression of Mast4 and chondrocyte marker genes in differentiating C3H10T1/2 cells treated with vactosertib. Vactosertib (0.5mM) was treated for 3 and 6 days in differentiating C3H10T1/2 cells. The mRNA expression of chondrocyte marker gene expression was examined by RT-PCR. The representative result were obtained from at least three separate experiments.

## Slide 15
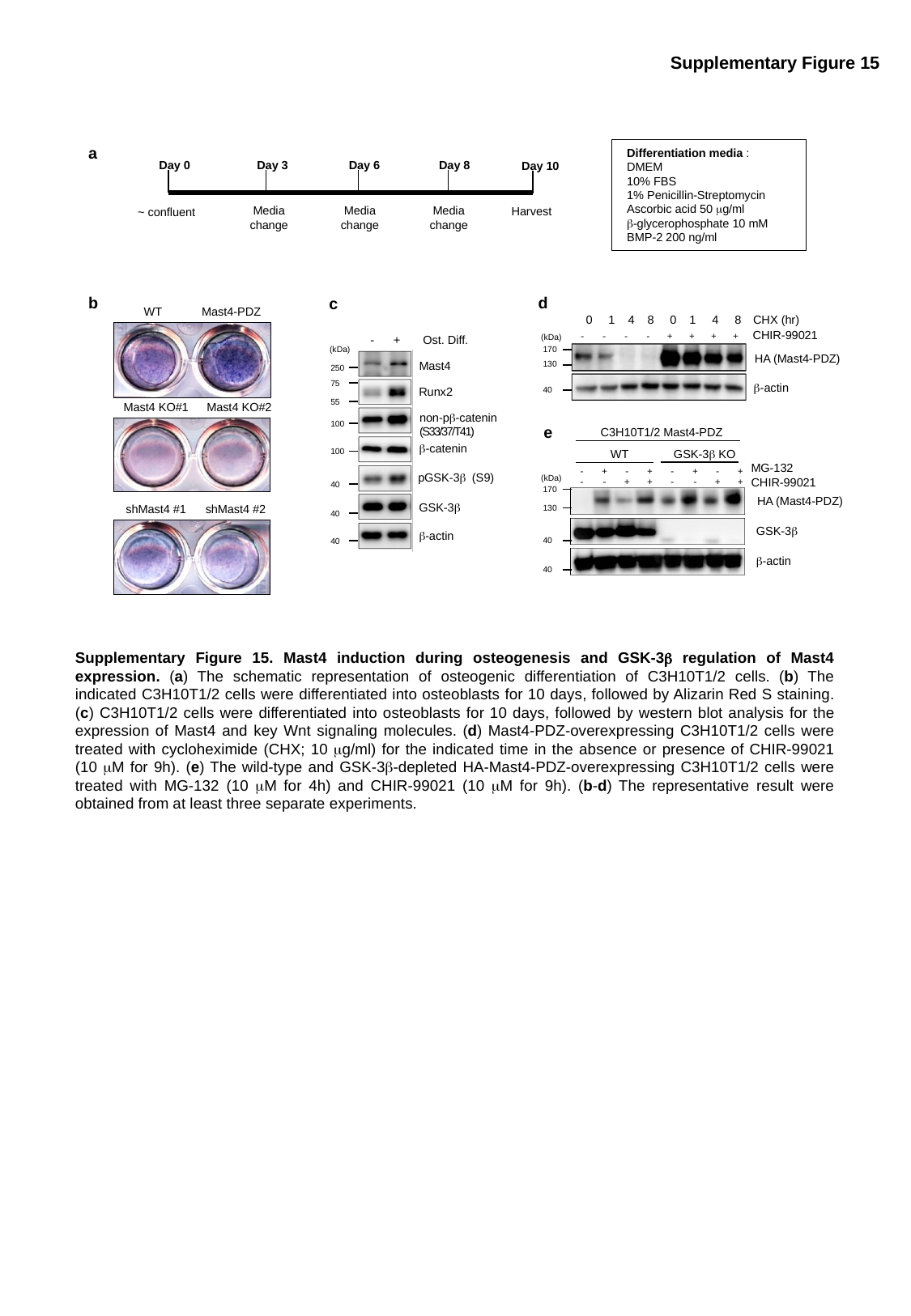

Supplementary Figure 15
a
 Differentiation media :
 DMEM
 10% FBS
 1% Penicillin-Streptomycin
 Ascorbic acid 50 mg/ml
 b-glycerophosphate 10 mM
 BMP-2 200 ng/ml
Day 0
Day 3
Day 6
Day 8
Day 10
Media change
Media change
Media change
Harvest
~ confluent
d
b
c
WT
Mast4-PDZ
Mast4 KO#1
Mast4 KO#2
shMast4 #1
shMast4 #2
CHX (hr)
0 1 4 8 0 1 4 8
CHIR-99021
- - - - + + + +
HA (Mast4-PDZ)
b-actin
(kDa)
- + Ost. Diff.
170
(kDa)
Mast4
130
250
75
40
Runx2
55
non-pb-catenin
(S33/37/T41)
100
e
C3H10T1/2 Mast4-PDZ
 WT GSK-3b KO
MG-132
CHIR-99021
- + - + - + - +
- - + + - - + +
HA (Mast4-PDZ)
GSK-3b
b-actin
b-catenin
100
pGSK-3b (S9)
(kDa)
40
170
GSK-3b
130
40
b-actin
40
40
40
Supplementary Figure 15. Mast4 induction during osteogenesis and GSK-3b regulation of Mast4 expression. (a) The schematic representation of osteogenic differentiation of C3H10T1/2 cells. (b) The indicated C3H10T1/2 cells were differentiated into osteoblasts for 10 days, followed by Alizarin Red S staining. (c) C3H10T1/2 cells were differentiated into osteoblasts for 10 days, followed by western blot analysis for the expression of Mast4 and key Wnt signaling molecules. (d) Mast4-PDZ-overexpressing C3H10T1/2 cells were treated with cycloheximide (CHX; 10 mg/ml) for the indicated time in the absence or presence of CHIR-99021 (10 mM for 9h). (e) The wild-type and GSK-3b-depleted HA-Mast4-PDZ-overexpressing C3H10T1/2 cells were treated with MG-132 (10 mM for 4h) and CHIR-99021 (10 mM for 9h). (b-d) The representative result were obtained from at least three separate experiments.

## Slide 16
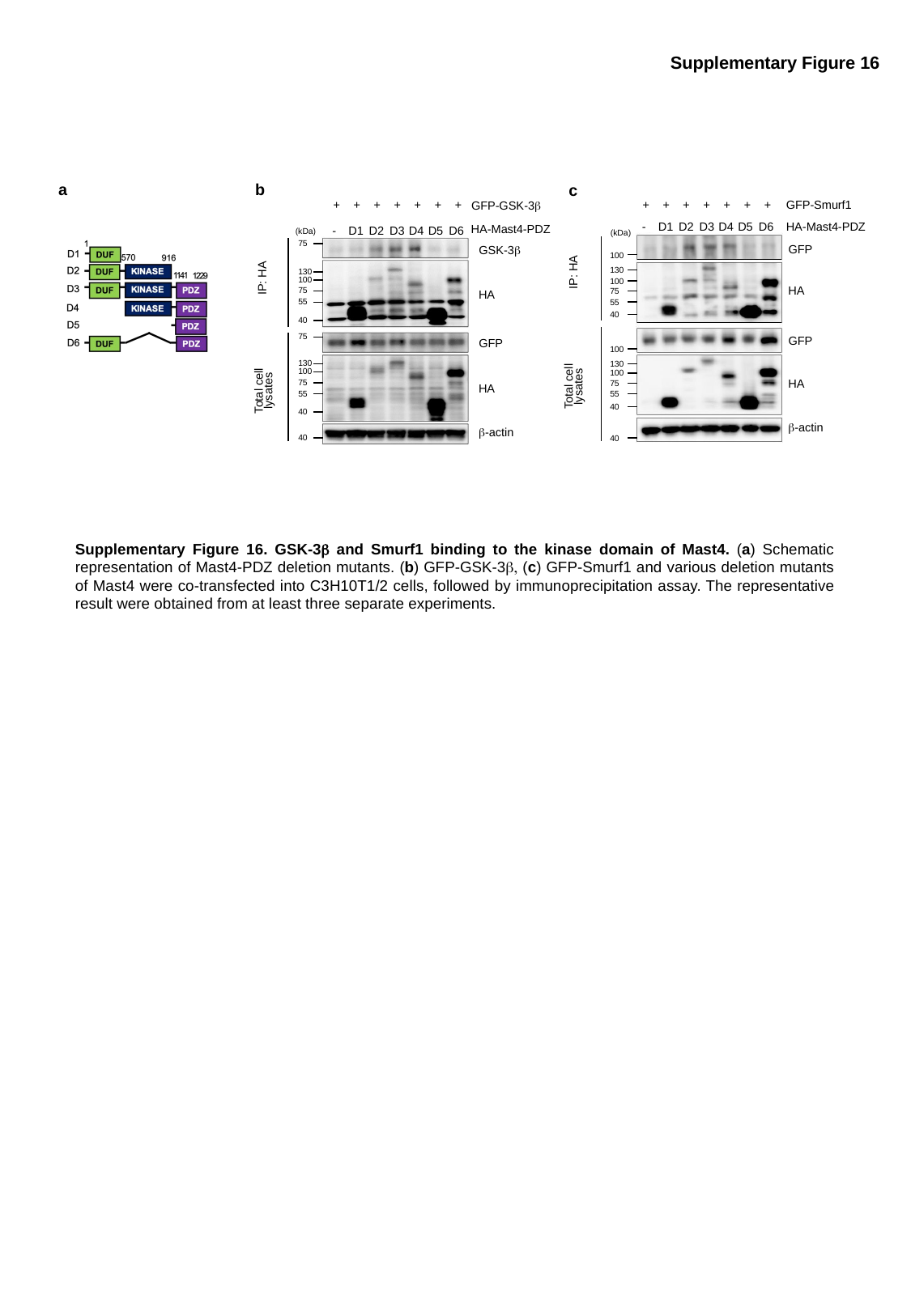

Supplementary Figure 16
a
b
c
GFP-Smurf1
+
+
+
+
+
+
+
+
+
+
+
+
+
+
GFP-GSK-3b
-
D1
D3
D4
D6
D2
D5
HA-Mast4-PDZ
HA-Mast4-PDZ
-
D1
D3
D4
D6
D2
D5
(kDa)
(kDa)
75
GFP
GSK-3b
100
IP: HA
130
130
IP: HA
100
100
HA
75
75
HA
55
55
40
40
75
GFP
GFP
100
Total cell lysates
Total cell lysates
130
130
100
100
HA
75
75
HA
55
55
40
40
b-actin
b-actin
40
40
Supplementary Figure 16. GSK-3b and Smurf1 binding to the kinase domain of Mast4. (a) Schematic representation of Mast4-PDZ deletion mutants. (b) GFP-GSK-3b, (c) GFP-Smurf1 and various deletion mutants of Mast4 were co-transfected into C3H10T1/2 cells, followed by immunoprecipitation assay. The representative result were obtained from at least three separate experiments.

## Slide 17
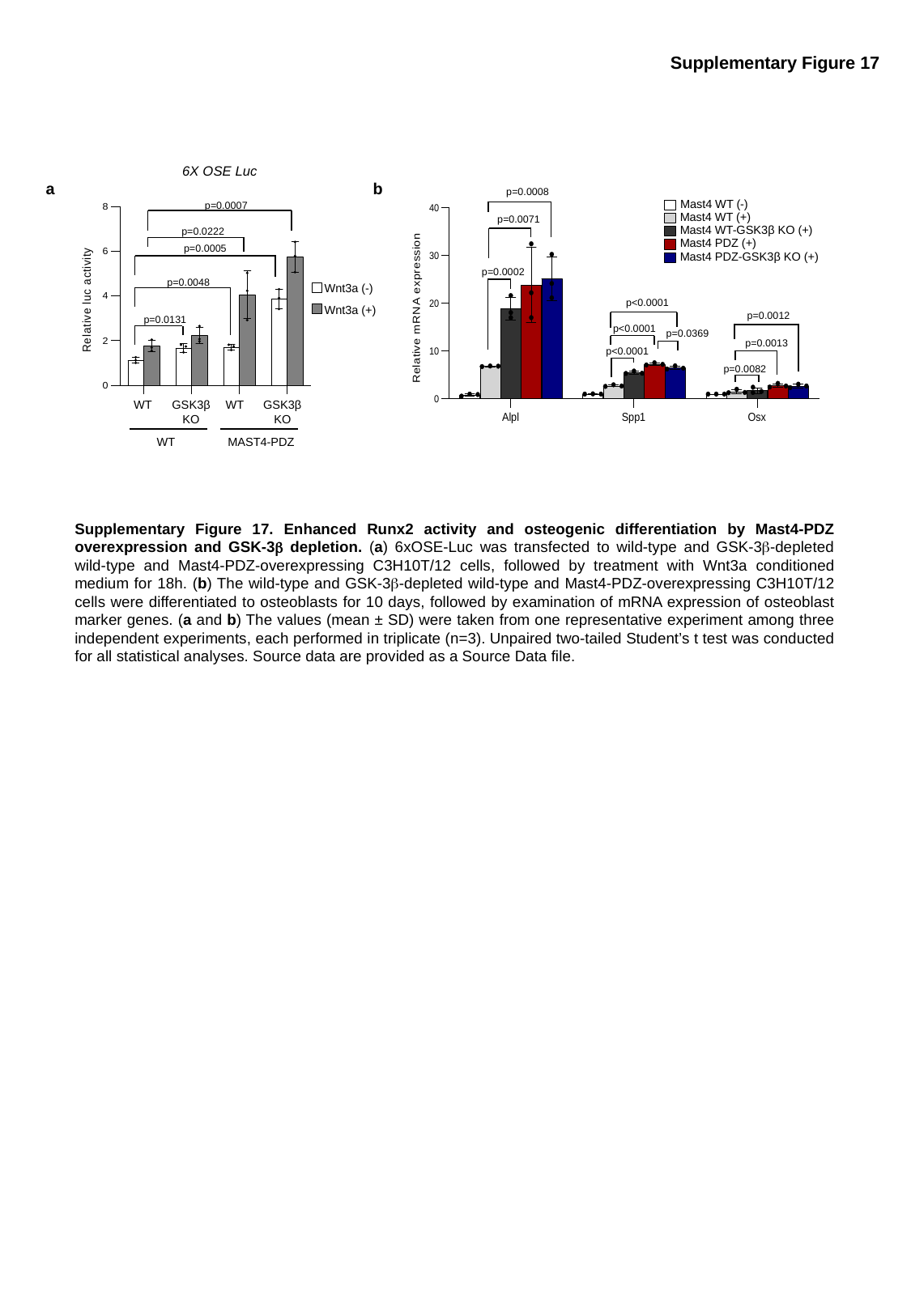

Supplementary Figure 17
6X OSE Luc
a
b
p=0.0008
Mast4 WT (-)
p=0.0007
Mast4 WT (+)
p=0.0071
Mast4 WT-GSK3β KO (+)
p=0.0222
Mast4 PDZ (+)
p=0.0005
Mast4 PDZ-GSK3β KO (+)
p=0.0002
p=0.0048
Wnt3a (-)
p<0.0001
Wnt3a (+)
p=0.0131
p=0.0012
p<0.0001
p=0.0369
p=0.0013
p<0.0001
p=0.0082
WT
GSK3β KO
WT
GSK3β KO
WT
MAST4-PDZ
Supplementary Figure 17. Enhanced Runx2 activity and osteogenic differentiation by Mast4-PDZ overexpression and GSK-3b depletion. (a) 6xOSE-Luc was transfected to wild-type and GSK-3b-depleted wild-type and Mast4-PDZ-overexpressing C3H10T/12 cells, followed by treatment with Wnt3a conditioned medium for 18h. (b) The wild-type and GSK-3b-depleted wild-type and Mast4-PDZ-overexpressing C3H10T/12 cells were differentiated to osteoblasts for 10 days, followed by examination of mRNA expression of osteoblast marker genes. (a and b) The values (mean ± SD) were taken from one representative experiment among three independent experiments, each performed in triplicate (n=3). Unpaired two-tailed Student’s t test was conducted for all statistical analyses. Source data are provided as a Source Data file.

## Slide 18
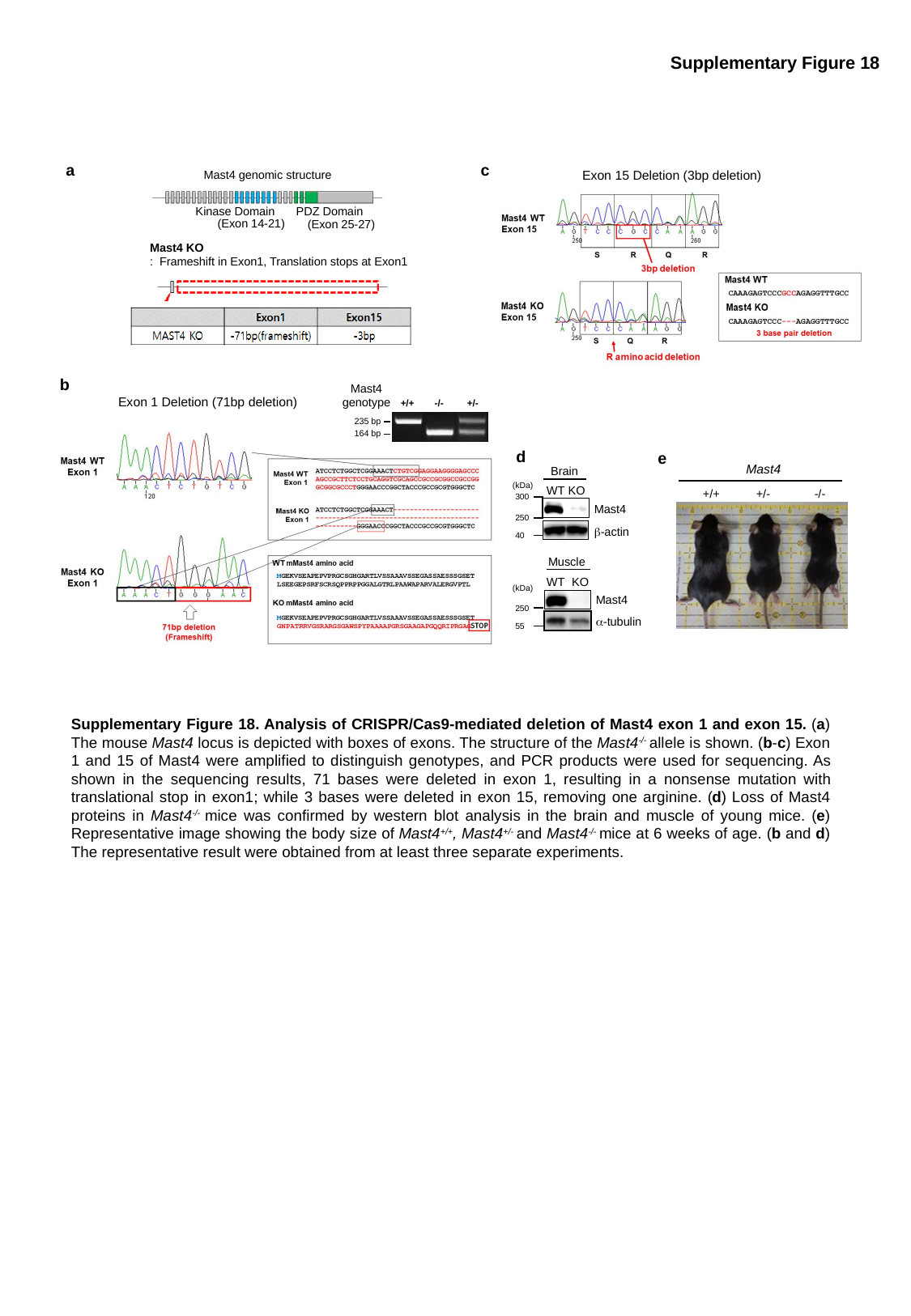

Supplementary Figure 18
a
c
Mast4 genomic structure
PDZ Domain
Kinase Domain
(Exon 14-21)
(Exon 25-27)
Mast4 KO
: Frameshift in Exon1, Translation stops at Exon1
Exon 15 Deletion (3bp deletion)
b
Mast4
genotype
+/-
+/+
-/-
235 bp
164 bp
Exon 1 Deletion (71bp deletion)
d
e
Mast4
+/+
+/-
-/-
Brain
WT
KO
Mast4
b-actin
Muscle
WT
KO
Mast4
a-tubulin
(kDa)
300
250
40
(kDa)
250
55
Supplementary Figure 18. Analysis of CRISPR/Cas9-mediated deletion of Mast4 exon 1 and exon 15. (a) The mouse Mast4 locus is depicted with boxes of exons. The structure of the Mast4-/- allele is shown. (b-c) Exon 1 and 15 of Mast4 were amplified to distinguish genotypes, and PCR products were used for sequencing. As shown in the sequencing results, 71 bases were deleted in exon 1, resulting in a nonsense mutation with translational stop in exon1; while 3 bases were deleted in exon 15, removing one arginine. (d) Loss of Mast4 proteins in Mast4-/- mice was confirmed by western blot analysis in the brain and muscle of young mice. (e) Representative image showing the body size of Mast4+/+, Mast4+/- and Mast4-/- mice at 6 weeks of age. (b and d) The representative result were obtained from at least three separate experiments.

## Slide 19
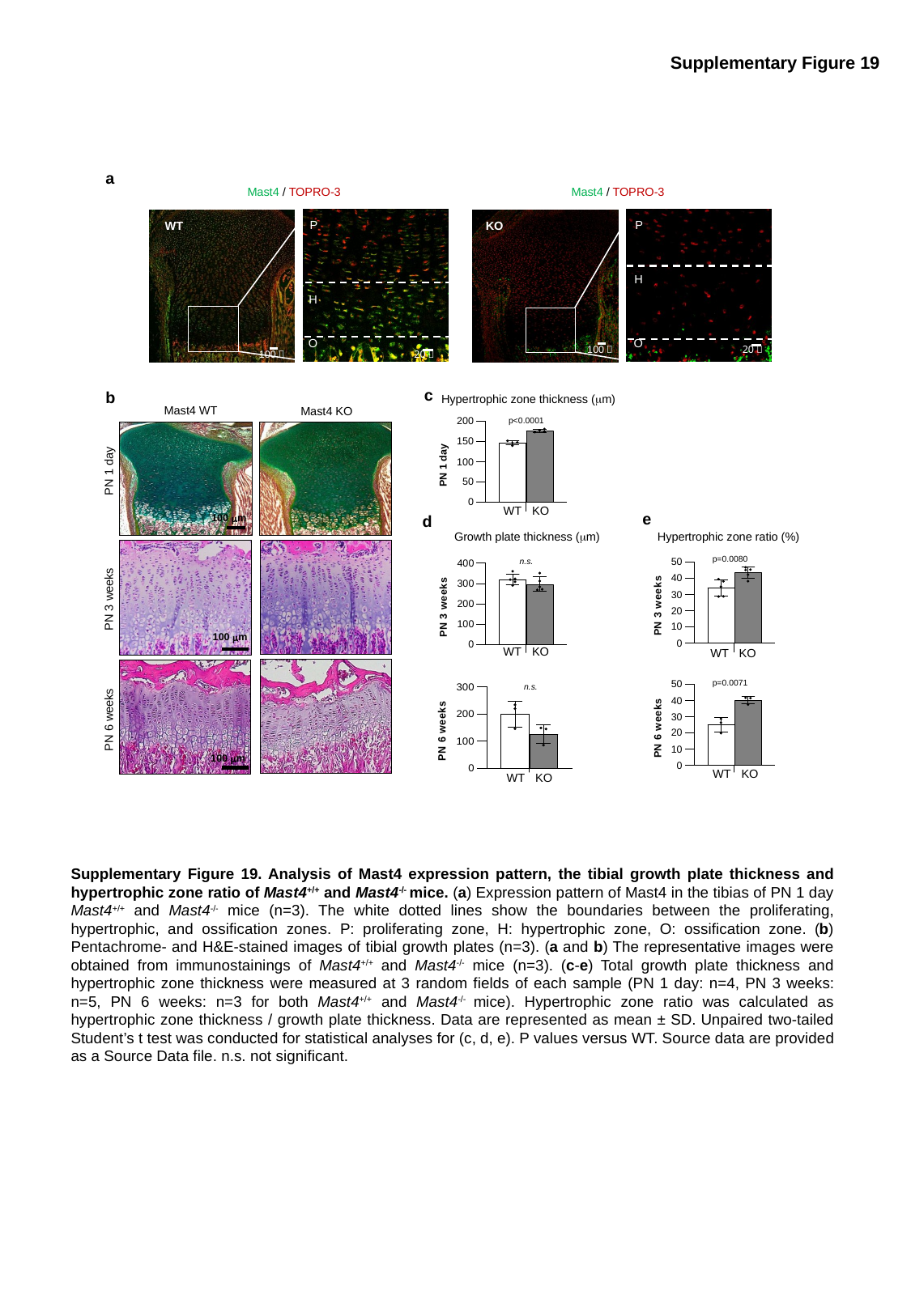

Supplementary Figure 19
a
Mast4 / TOPRO-3
Mast4 / TOPRO-3
P
P
WT
KO
H
H
O
O
20㎛
100㎛
20㎛
100㎛
c
b
Hypertrophic zone thickness (mm)
Mast4 WT
Mast4 KO
p<0.0001
100 mm
100 mm
100 mm
PN 1 day
WT
KO
e
d
Growth plate thickness (mm)
Hypertrophic zone ratio (%)
WT
KO
p=0.0080
n.s.
PN 3 weeks
WT
KO
p=0.0071
n.s.
PN 6 weeks
WT
KO
WT
KO
Supplementary Figure 19. Analysis of Mast4 expression pattern, the tibial growth plate thickness and hypertrophic zone ratio of Mast4+/+ and Mast4-/- mice. (a) Expression pattern of Mast4 in the tibias of PN 1 day Mast4+/+ and Mast4-/- mice (n=3). The white dotted lines show the boundaries between the proliferating, hypertrophic, and ossification zones. P: proliferating zone, H: hypertrophic zone, O: ossification zone. (b) Pentachrome- and H&E-stained images of tibial growth plates (n=3). (a and b) The representative images were obtained from immunostainings of Mast4+/+ and Mast4-/- mice (n=3). (c-e) Total growth plate thickness and hypertrophic zone thickness were measured at 3 random fields of each sample (PN 1 day: n=4, PN 3 weeks: n=5, PN 6 weeks: n=3 for both Mast4+/+ and Mast4-/- mice). Hypertrophic zone ratio was calculated as hypertrophic zone thickness / growth plate thickness. Data are represented as mean ± SD. Unpaired two-tailed Student’s t test was conducted for statistical analyses for (c, d, e). P values versus WT. Source data are provided as a Source Data file. n.s. not significant.

## Slide 20
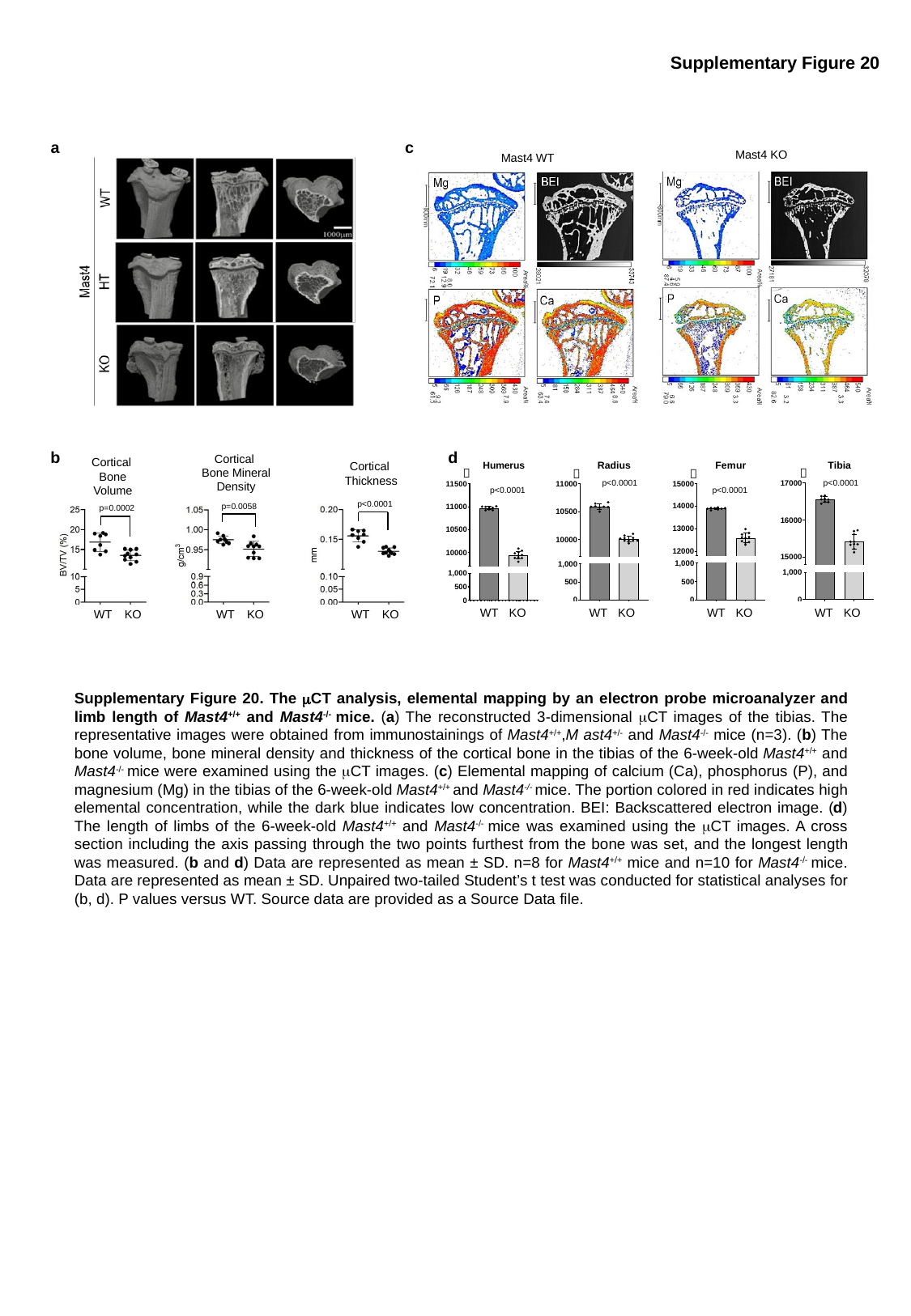

Supplementary Figure 20
c
a
Mast4 KO
Mast4 WT
d
b
Cortical
Bone Mineral Density
Cortical
Bone Volume
Cortical
Thickness
p<0.0001
p=0.0058
p=0.0002
WT
KO
WT
KO
WT
p<0.0001
p<0.0001
p<0.0001
p<0.0001
WT
KO
WT
KO
WT
KO
WT
KO
WT
KO
WT
KO
WT
KO
Supplementary Figure 20. The mCT analysis, elemental mapping by an electron probe microanalyzer and limb length of Mast4+/+ and Mast4-/- mice. (a) The reconstructed 3-dimensional mCT images of the tibias. The representative images were obtained from immunostainings of Mast4+/+,M ast4+/- and Mast4-/- mice (n=3). (b) The bone volume, bone mineral density and thickness of the cortical bone in the tibias of the 6-week-old Mast4+/+ and Mast4-/- mice were examined using the mCT images. (c) Elemental mapping of calcium (Ca), phosphorus (P), and magnesium (Mg) in the tibias of the 6-week-old Mast4+/+ and Mast4-/- mice. The portion colored in red indicates high elemental concentration, while the dark blue indicates low concentration. BEI: Backscattered electron image. (d) The length of limbs of the 6-week-old Mast4+/+ and Mast4-/- mice was examined using the mCT images. A cross section including the axis passing through the two points furthest from the bone was set, and the longest length was measured. (b and d) Data are represented as mean ± SD. n=8 for Mast4+/+ mice and n=10 for Mast4-/- mice. Data are represented as mean ± SD. Unpaired two-tailed Student’s t test was conducted for statistical analyses for (b, d). P values versus WT. Source data are provided as a Source Data file.

## Slide 21
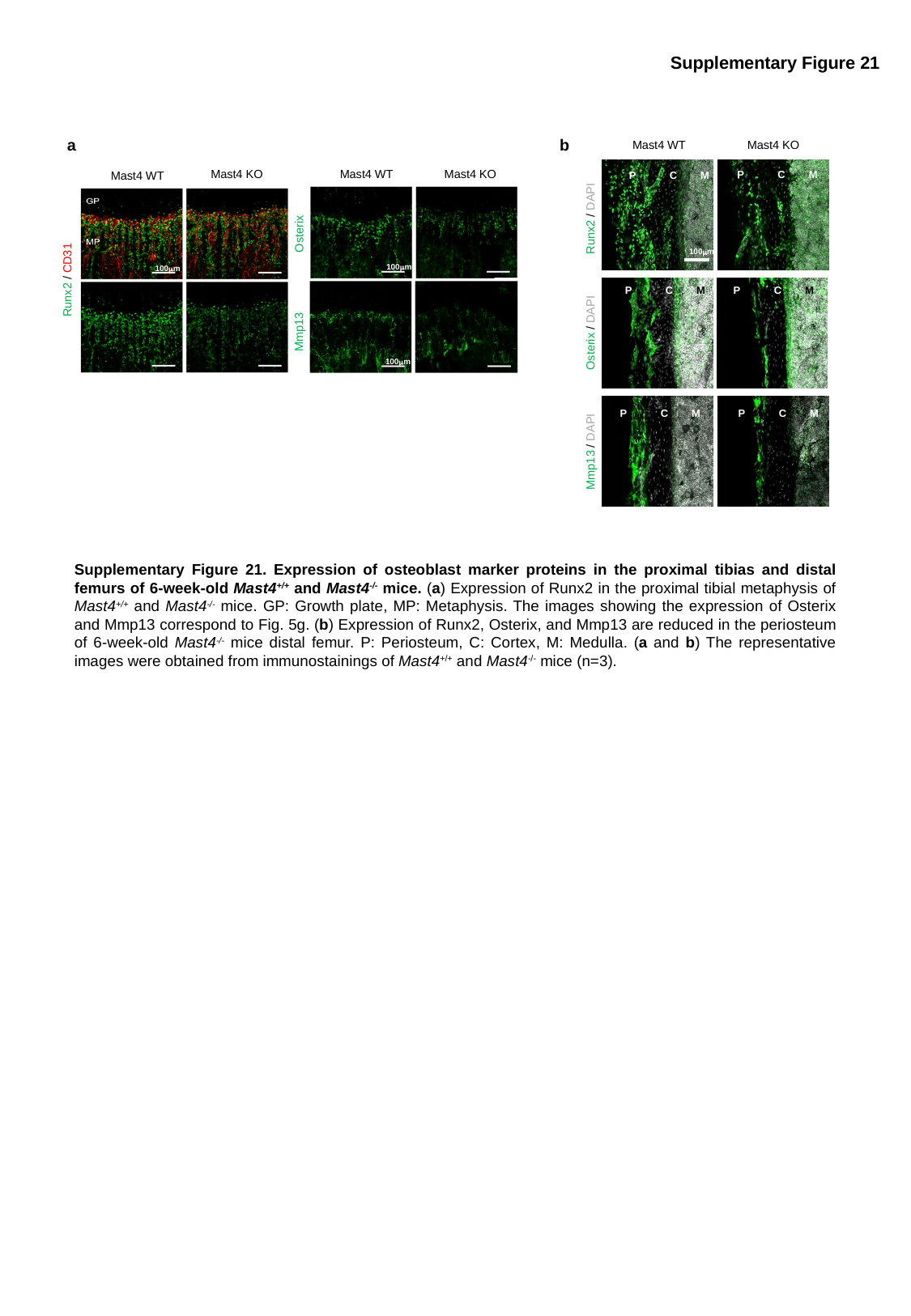

Supplementary Figure 21
a
b
Mast4 WT
Mast4 KO
P
C
M
P
C
M
Runx2 / DAPI
100mm
P
C
M
P
C
M
Osterix / DAPI
P
C
P
C
M
M
Mmp13 / DAPI
Mast4 KO
Mast4 WT
Mast4 KO
100mm
Osterix
100mm
Mmp13
100mm
Mast4 WT
Runx2 / CD31
100mm
Supplementary Figure 21. Expression of osteoblast marker proteins in the proximal tibias and distal femurs of 6-week-old Mast4+/+ and Mast4-/- mice. (a) Expression of Runx2 in the proximal tibial metaphysis of Mast4+/+ and Mast4-/- mice. GP: Growth plate, MP: Metaphysis. The images showing the expression of Osterix and Mmp13 correspond to Fig. 5g. (b) Expression of Runx2, Osterix, and Mmp13 are reduced in the periosteum of 6-week-old Mast4-/- mice distal femur. P: Periosteum, C: Cortex, M: Medulla. (a and b) The representative images were obtained from immunostainings of Mast4+/+ and Mast4-/- mice (n=3).

## Slide 22
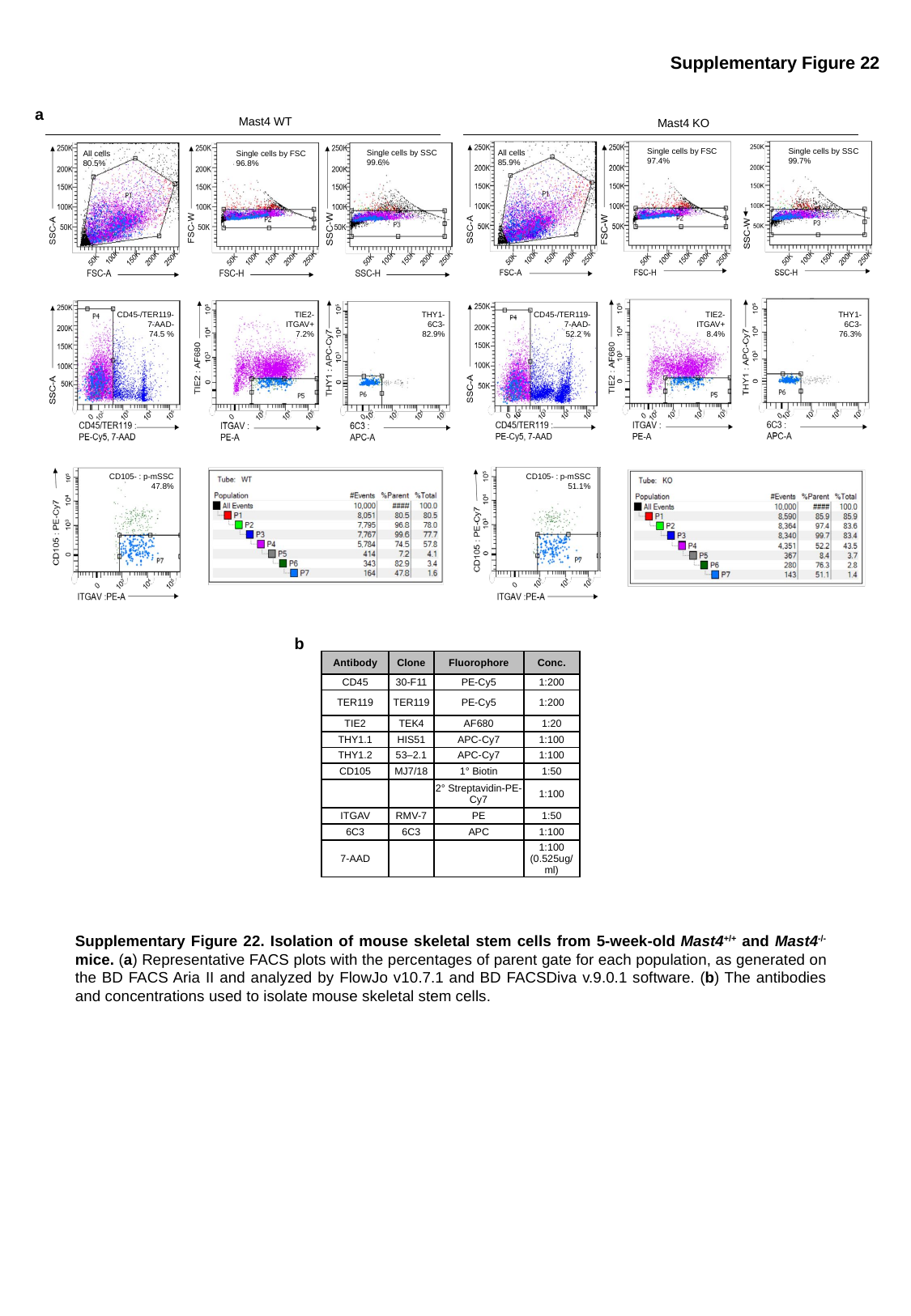

Supplementary Figure 22
a
Mast4 WT
Mast4 KO
Single cells by FSC
97.4%
Single cells by SSC
99.7%
Single cells by SSC
99.6%
Single cells by FSC
96.8%
All cells
85.9%
All cells
80.5%
THY1-
6C3-
82.9%
THY1-
6C3-
76.3%
CD45-/TER119-
7-AAD-
52.2 %
TIE2-
ITGAV+
7.2%
TIE2-
ITGAV+
8.4%
CD45-/TER119-
7-AAD-
74.5 %
CD105- : p-mSSC
47.8%
CD105- : p-mSSC
51.1%
b
| Antibody | Clone | Fluorophore | Conc. |
| --- | --- | --- | --- |
| CD45 | 30-F11 | PE-Cy5 | 1:200 |
| TER119 | TER119 | PE-Cy5 | 1:200 |
| TIE2 | TEK4 | AF680 | 1:20 |
| THY1.1 | HIS51 | APC-Cy7 | 1:100 |
| THY1.2 | 53–2.1 | APC-Cy7 | 1:100 |
| CD105 | MJ7/18 | 1° Biotin | 1:50 |
| | | 2° Streptavidin-PE-Cy7 | 1:100 |
| ITGAV | RMV-7 | PE | 1:50 |
| 6C3 | 6C3 | APC | 1:100 |
| 7-AAD | | | 1:100 (0.525ug/ml) |
Supplementary Figure 22. Isolation of mouse skeletal stem cells from 5-week-old Mast4+/+ and Mast4-/- mice. (a) Representative FACS plots with the percentages of parent gate for each population, as generated on the BD FACS Aria II and analyzed by FlowJo v10.7.1 and BD FACSDiva v.9.0.1 software. (b) The antibodies and concentrations used to isolate mouse skeletal stem cells.

## Slide 23
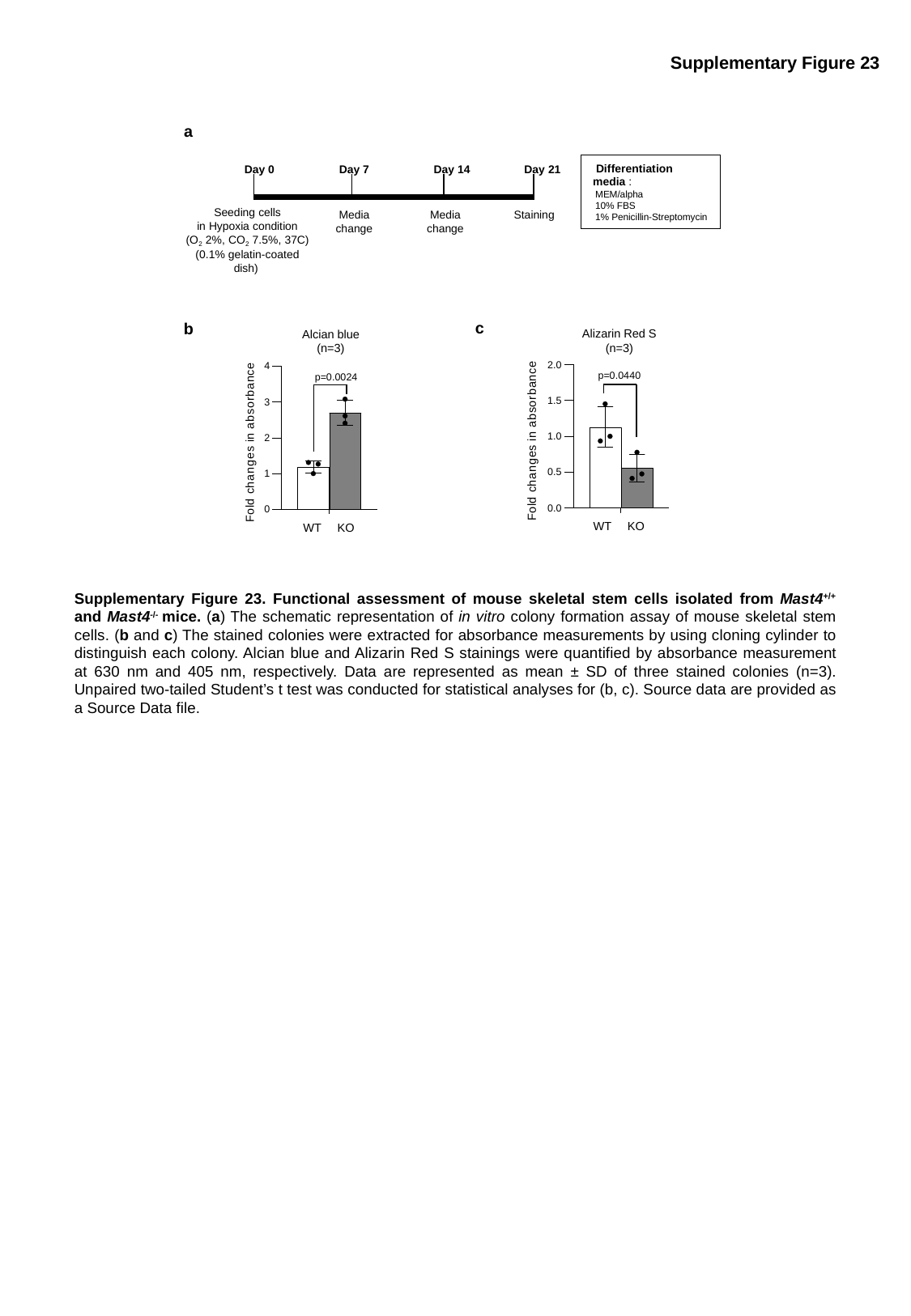

Supplementary Figure 23
a
 Differentiation media :
 MEM/alpha
 10% FBS
 1% Penicillin-Streptomycin
Day 0
Day 7
Day 14
Day 21
Seeding cells
in Hypoxia condition
(O2 2%, CO2 7.5%, 37C)
(0.1% gelatin-coated dish)
Media change
Media change
Staining
c
b
Alizarin Red S
(n=3)
Alcian blue
(n=3)
p=0.0024
p=0.0440
WT KO
WT KO
Supplementary Figure 23. Functional assessment of mouse skeletal stem cells isolated from Mast4+/+ and Mast4-/- mice. (a) The schematic representation of in vitro colony formation assay of mouse skeletal stem cells. (b and c) The stained colonies were extracted for absorbance measurements by using cloning cylinder to distinguish each colony. Alcian blue and Alizarin Red S stainings were quantified by absorbance measurement at 630 nm and 405 nm, respectively. Data are represented as mean ± SD of three stained colonies (n=3). Unpaired two-tailed Student’s t test was conducted for statistical analyses for (b, c). Source data are provided as a Source Data file.

## Slide 24
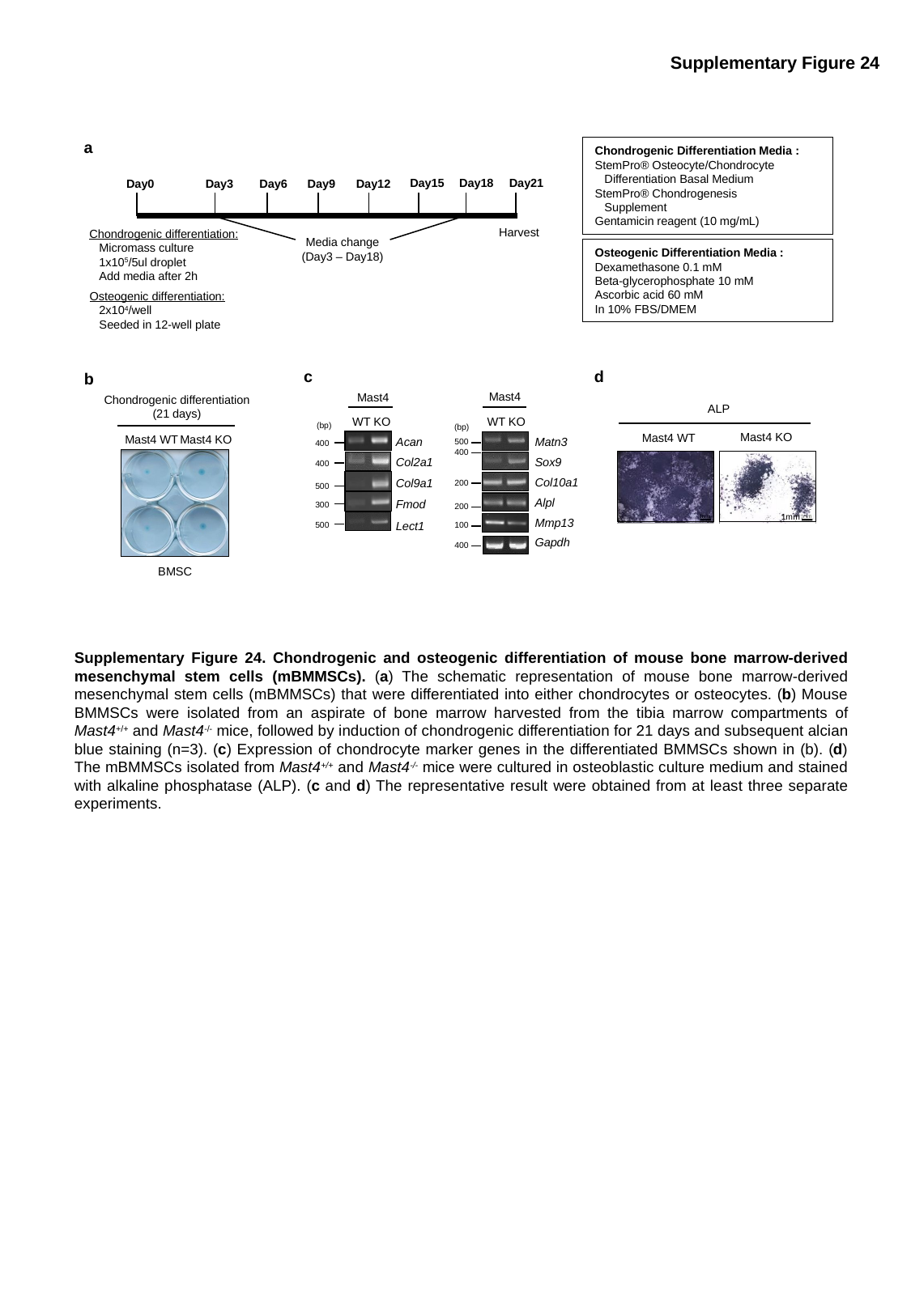

Supplementary Figure 24
a
Chondrogenic Differentiation Media :
StemPro® Osteocyte/Chondrocyte
 Differentiation Basal Medium
StemPro® Chondrogenesis
 Supplement
Gentamicin reagent (10 mg/mL)
Day18
Day21
Day15
Day0
Day3
Day9
Day12
Day6
Harvest
Chondrogenic differentiation:
 Micromass culture
 1x105/5ul droplet
 Add media after 2h
Media change
(Day3 – Day18)
Osteogenic differentiation:
 2x10^4^/well
 Seeded in 12-well plate
Osteogenic Differentiation Media :
Dexamethasone 0.1 mM
Beta-glycerophosphate 10 mM
Ascorbic acid 60 mM
In 10% FBS/DMEM
d
c
b
Mast4
Mast4
Chondrogenic differentiation
(21 days)
Mast4 WT
Mast4 KO
BMSC
ALP
Mast4 KO
Mast4 WT
1mm
WT
KO
WT
KO
(bp)
(bp)
Matn3
Acan
500
400
400
Col2a1
Sox9
400
Col10a1
Col9a1
200
500
Alpl
Fmod
300
200
Mmp13
Lect1
500
100
Gapdh
400
Supplementary Figure 24. Chondrogenic and osteogenic differentiation of mouse bone marrow-derived mesenchymal stem cells (mBMMSCs). (a) The schematic representation of mouse bone marrow-derived mesenchymal stem cells (mBMMSCs) that were differentiated into either chondrocytes or osteocytes. (b) Mouse BMMSCs were isolated from an aspirate of bone marrow harvested from the tibia marrow compartments of Mast4+/+ and Mast4-/- mice, followed by induction of chondrogenic differentiation for 21 days and subsequent alcian blue staining (n=3). (c) Expression of chondrocyte marker genes in the differentiated BMMSCs shown in (b). (d) The mBMMSCs isolated from Mast4+/+ and Mast4-/- mice were cultured in osteoblastic culture medium and stained with alkaline phosphatase (ALP). (c and d) The representative result were obtained from at least three separate experiments.

## Slide 25
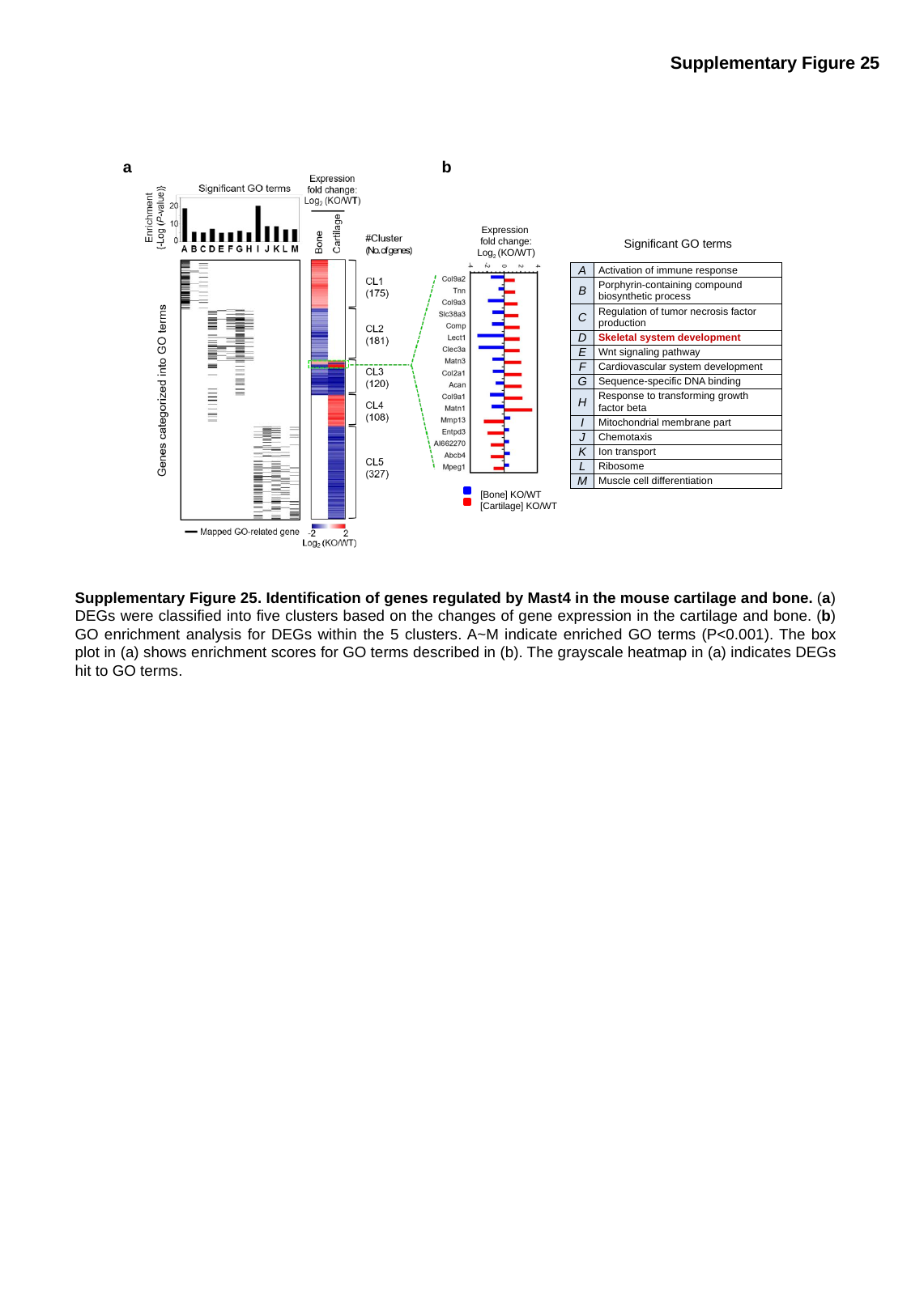

Supplementary Figure 25
a
b
Expression
fold change:
Log2 (KO/WT)
[Bone] KO/WT
[Cartilage] KO/WT
Significant GO terms
| A | Activation of immune response |
| --- | --- |
| B | Porphyrin-containing compound biosynthetic process |
| C | Regulation of tumor necrosis factor production |
| D | Skeletal system development |
| E | Wnt signaling pathway |
| F | Cardiovascular system development |
| G | Sequence-specific DNA binding |
| H | Response to transforming growth factor beta |
| I | Mitochondrial membrane part |
| J | Chemotaxis |
| K | Ion transport |
| L | Ribosome |
| M | Muscle cell differentiation |
Supplementary Figure 25. Identification of genes regulated by Mast4 in the mouse cartilage and bone. (a) DEGs were classified into five clusters based on the changes of gene expression in the cartilage and bone. (b) GO enrichment analysis for DEGs within the 5 clusters. A~M indicate enriched GO terms (P<0.001). The box plot in (a) shows enrichment scores for GO terms described in (b). The grayscale heatmap in (a) indicates DEGs hit to GO terms.

## Slide 26
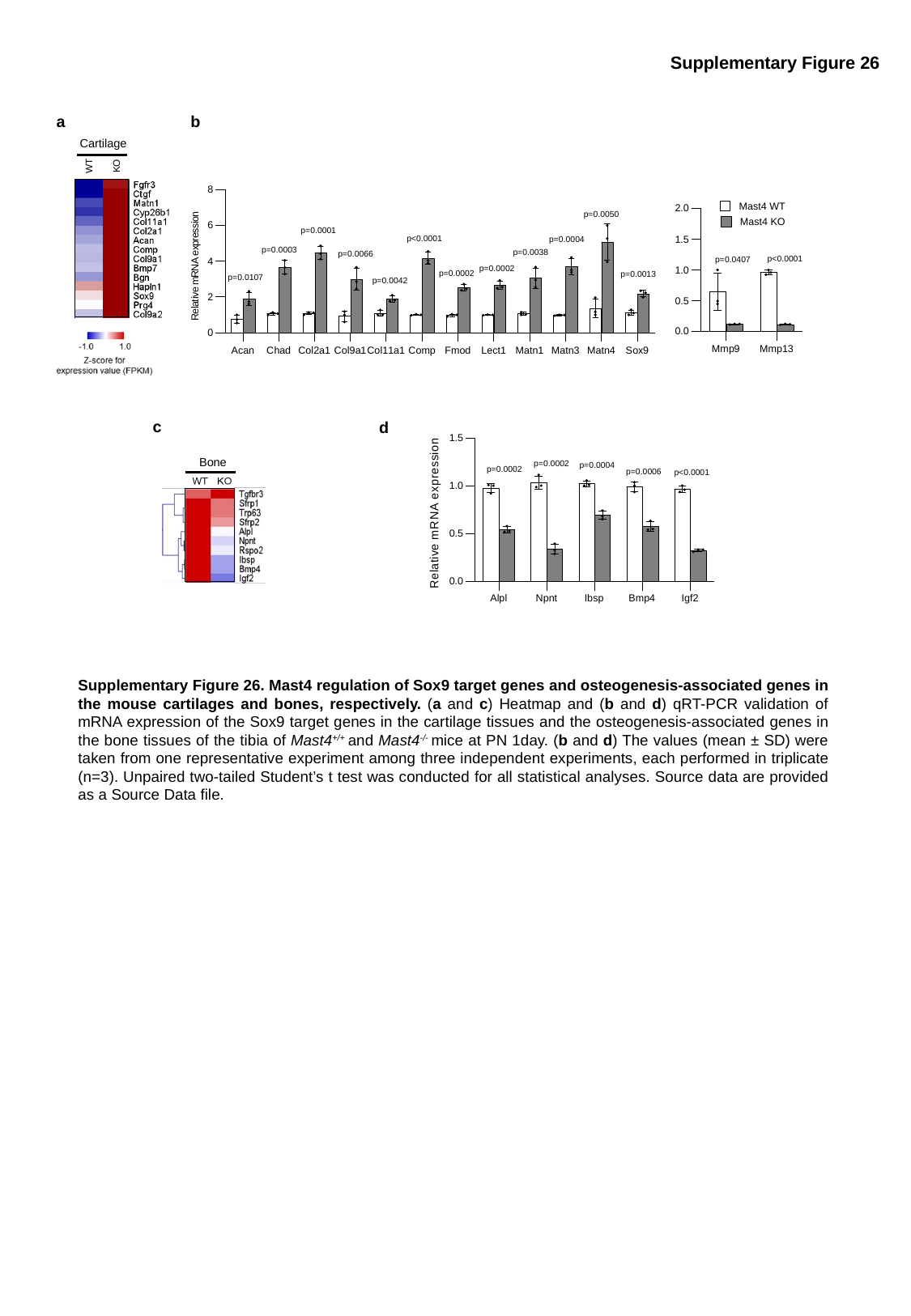

Supplementary Figure 26
a
b
Cartilage
WT
KO
Mast4 WT
p=0.0050
Mast4 KO
p=0.0001
p<0.0001
p=0.0004
p=0.0003
p=0.0038
p=0.0066
p<0.0001
p=0.0407
p=0.0002
p=0.0002
p=0.0013
p=0.0107
p=0.0042
c
d
Bone
p=0.0002
p=0.0004
p=0.0002
p=0.0006
p<0.0001
Supplementary Figure 26. Mast4 regulation of Sox9 target genes and osteogenesis-associated genes in the mouse cartilages and bones, respectively. (a and c) Heatmap and (b and d) qRT-PCR validation of mRNA expression of the Sox9 target genes in the cartilage tissues and the osteogenesis-associated genes in the bone tissues of the tibia of Mast4+/+ and Mast4-/- mice at PN 1day. (b and d) The values (mean ± SD) were taken from one representative experiment among three independent experiments, each performed in triplicate (n=3). Unpaired two-tailed Student’s t test was conducted for all statistical analyses. Source data are provided as a Source Data file.

## Slide 27
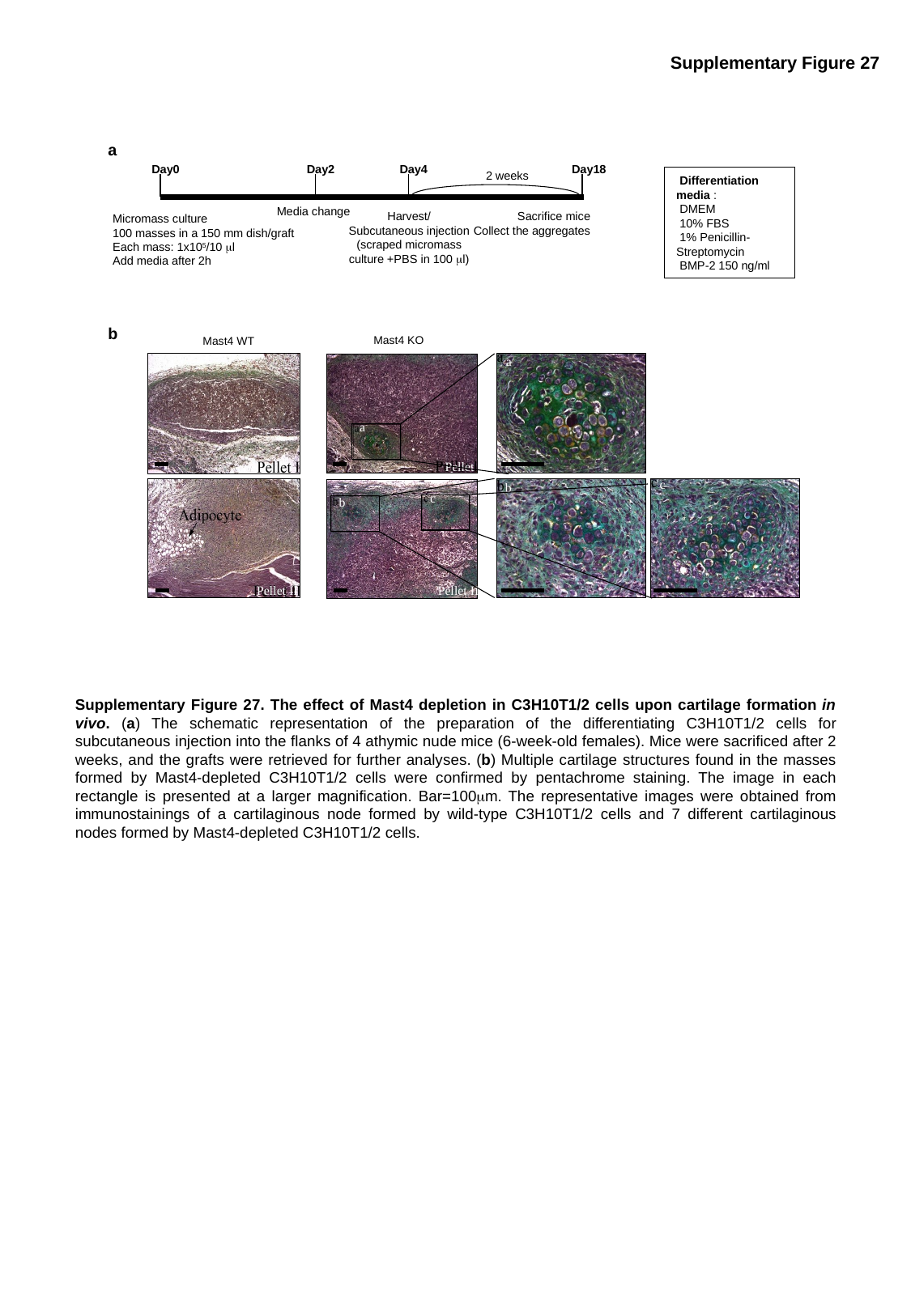

Supplementary Figure 27
a
Day0
Day2
Day4
Day18
2 weeks
 Differentiation media :
 DMEM
 10% FBS
 1% Penicillin-Streptomycin
 BMP-2 150 ng/ml
Media change
Sacrifice mice
Collect the aggregates
Harvest/
Subcutaneous injection
(scraped micromass culture +PBS in 100 ml)
Micromass culture
100 masses in a 150 mm dish/graft
Each mass: 1x105/10 ml
Add media after 2h
b
Mast4 KO
Mast4 WT
a
a
Pellet I
c
b
c
b
Pellet II
Pellet II
Supplementary Figure 27. The effect of Mast4 depletion in C3H10T1/2 cells upon cartilage formation in vivo. (a) The schematic representation of the preparation of the differentiating C3H10T1/2 cells for subcutaneous injection into the flanks of 4 athymic nude mice (6-week-old females). Mice were sacrificed after 2 weeks, and the grafts were retrieved for further analyses. (b) Multiple cartilage structures found in the masses formed by Mast4-depleted C3H10T1/2 cells were confirmed by pentachrome staining. The image in each rectangle is presented at a larger magnification. Bar=100mm. The representative images were obtained from immunostainings of a cartilaginous node formed by wild-type C3H10T1/2 cells and 7 different cartilaginous nodes formed by Mast4-depleted C3H10T1/2 cells.

## Slide 28
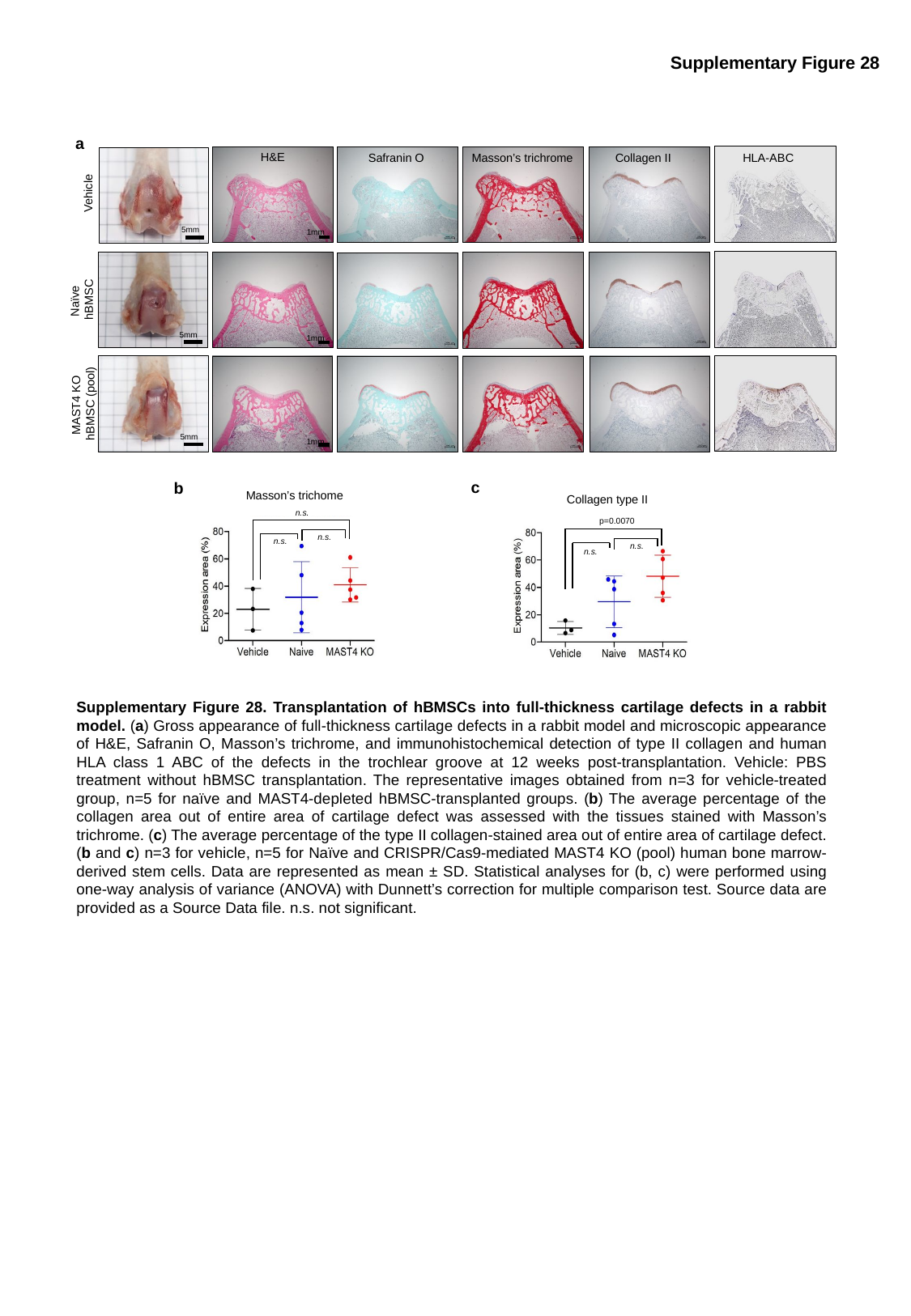

Supplementary Figure 28
a
H&E
Masson’s trichrome
HLA-ABC
Collagen II
Safranin O
1mm
H&E
5mm
Vehicle
5mm
1mm
Naïve
hBMSC
5mm
1mm
MAST4 KO
hBMSC (pool)
c
b
Masson’s trichome
Collagen type II
n.s.
p=0.0070
n.s.
n.s.
n.s.
n.s.
Supplementary Figure 28. Transplantation of hBMSCs into full-thickness cartilage defects in a rabbit model. (a) Gross appearance of full-thickness cartilage defects in a rabbit model and microscopic appearance of H&E, Safranin O, Masson’s trichrome, and immunohistochemical detection of type II collagen and human HLA class 1 ABC of the defects in the trochlear groove at 12 weeks post-transplantation. Vehicle: PBS treatment without hBMSC transplantation. The representative images obtained from n=3 for vehicle-treated group, n=5 for naïve and MAST4-depleted hBMSC-transplanted groups. (b) The average percentage of the collagen area out of entire area of cartilage defect was assessed with the tissues stained with Masson’s trichrome. (c) The average percentage of the type II collagen-stained area out of entire area of cartilage defect. (b and c) n=3 for vehicle, n=5 for Naïve and CRISPR/Cas9-mediated MAST4 KO (pool) human bone marrow-derived stem cells. Data are represented as mean ± SD. Statistical analyses for (b, c) were performed using one-way analysis of variance (ANOVA) with Dunnett’s correction for multiple comparison test. Source data are provided as a Source Data file. n.s. not significant.

## Slide 29
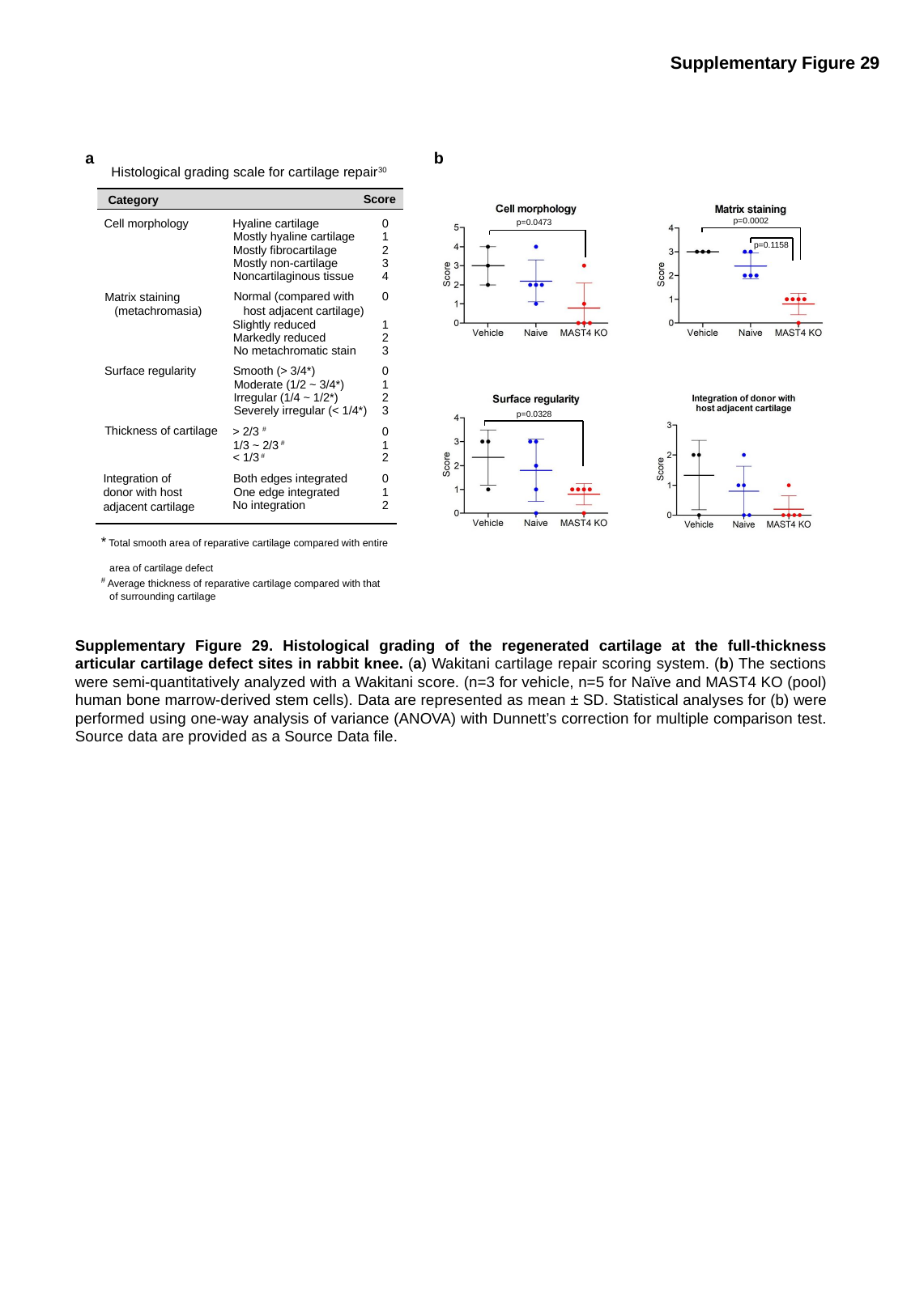

Supplementary Figure 29
b
a
Histological grading scale for cartilage repair30
Score
Category
p=0.0002
Cell morphology
Hyaline cartilage
0
p=0.0473
Mostly hyaline cartilage
1
p=0.1158
Mostly fibrocartilage
2
Mostly non-cartilage
3
Noncartilaginous tissue
4
Normal (compared with
 host adjacent cartilage)
0
Matrix staining
 (metachromasia)
Slightly reduced
1
Markedly reduced
2
No metachromatic stain
3
Surface regularity
Smooth (> 3/4*)
0
Moderate (1/2 ~ 3/4*)
1
Irregular (1/4 ~ 1/2*)
2
Severely irregular (< 1/4*)
3
p=0.0328
Thickness of cartilage
> 2/3 #
0
1/3 ~ 2/3 #
1
< 1/3 #
2
Integration of donor with host adjacent cartilage
Both edges integrated
0
One edge integrated
1
No integration
2
* Total smooth area of reparative cartilage compared with entire
 area of cartilage defect
# Average thickness of reparative cartilage compared with that
 of surrounding cartilage
Supplementary Figure 29. Histological grading of the regenerated cartilage at the full-thickness articular cartilage defect sites in rabbit knee. (a) Wakitani cartilage repair scoring system. (b) The sections were semi-quantitatively analyzed with a Wakitani score. (n=3 for vehicle, n=5 for Naïve and MAST4 KO (pool) human bone marrow-derived stem cells). Data are represented as mean ± SD. Statistical analyses for (b) were performed using one-way analysis of variance (ANOVA) with Dunnett’s correction for multiple comparison test. Source data are provided as a Source Data file.

## Slide 30
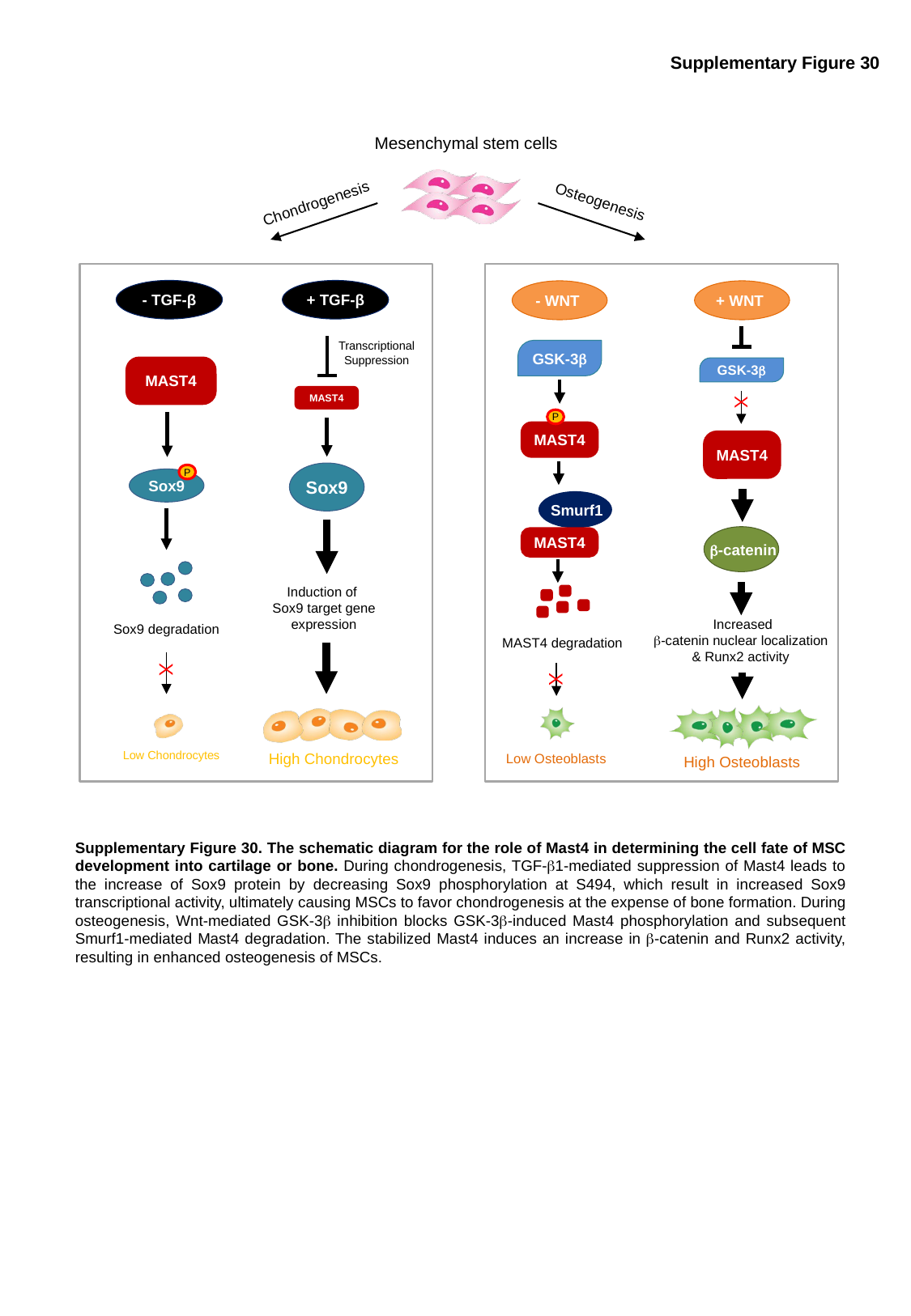

Supplementary Figure 30
Mesenchymal stem cells
Osteogenesis
Chondrogenesis
- TGF-β
+ TGF-β
- WNT
+ WNT
Transcriptional
Suppression
GSK-3b
GSK-3b
MAST4
X
MAST4
P
MAST4
MAST4
Sox9
P
Sox9
Smurf1
b-catenin
MAST4
Induction of
Sox9 target gene expression
Increased
b-catenin nuclear localization
& Runx2 activity
Sox9 degradation
MAST4 degradation
X
X
High Osteoblasts
High Chondrocytes
Low Chondrocytes
Low Osteoblasts
Supplementary Figure 30. The schematic diagram for the role of Mast4 in determining the cell fate of MSC development into cartilage or bone. During chondrogenesis, TGF-β1-mediated suppression of Mast4 leads to the increase of Sox9 protein by decreasing Sox9 phosphorylation at S494, which result in increased Sox9 transcriptional activity, ultimately causing MSCs to favor chondrogenesis at the expense of bone formation. During osteogenesis, Wnt-mediated GSK-3b inhibition blocks GSK-3b-induced Mast4 phosphorylation and subsequent Smurf1-mediated Mast4 degradation. The stabilized Mast4 induces an increase in b-catenin and Runx2 activity, resulting in enhanced osteogenesis of MSCs.

## Slide 31
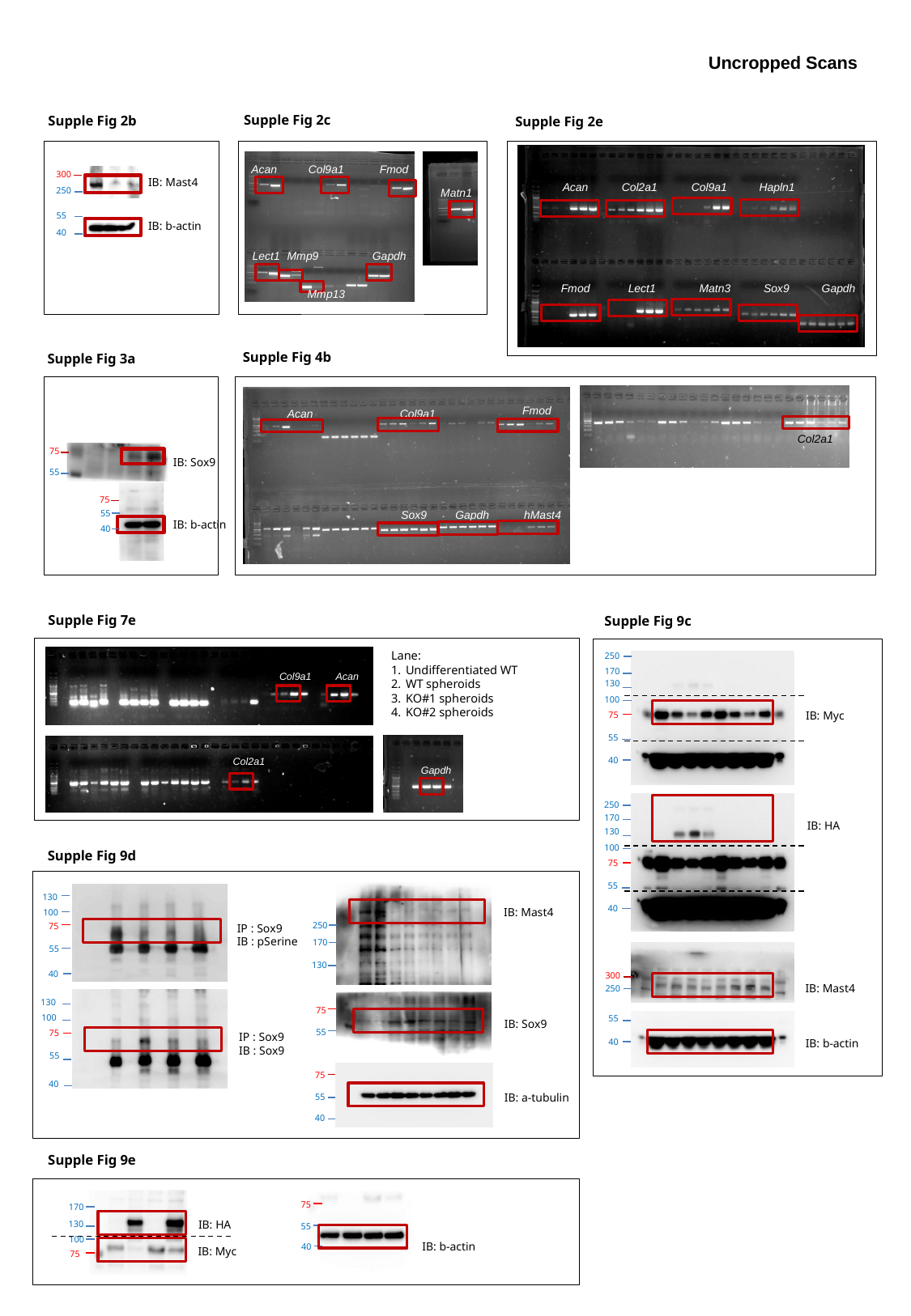

Uncropped Scans
Supple Fig 2c
Supple Fig 2b
Supple Fig 2e
300
IB: Mast4
250
55
IB: b-actin
40
Acan
Col2a1
Col9a1
Hapln1
Fmod
Lect1
Matn3
Sox9
Gapdh
Acan
Col9a1
Fmod
Matn1
Lect1
Mmp9
Gapdh
Mmp13
Supple Fig 4b
Supple Fig 3a
Fmod
Col9a1
Acan
Col2a1
hMast4
Gapdh
Sox9
75
IB: Sox9
55
75
55
IB: b-actin
40
Supple Fig 7e
Supple Fig 9c
Lane:
Undifferentiated WT
WT spheroids
KO#1 spheroids
KO#2 spheroids
Col9a1
Acan
Col2a1
Gapdh
250
170
130
100
IB: Myc
75
55
40
250
170
IB: HA
130
100
75
55
40
300
IB: Mast4
250
55
40
IB: b-actin
Supple Fig 9d
130
IB: Mast4
100
250
75
IP : Sox9
IB : pSerine
170
55
130
40
130
75
100
IB: Sox9
55
75
IP : Sox9
IB : Sox9
55
75
40
IB: a-tubulin
55
40
Supple Fig 9e
75
170
IB: HA
130
55
100
IB: b-actin
40
IB: Myc
75

## Slide 32
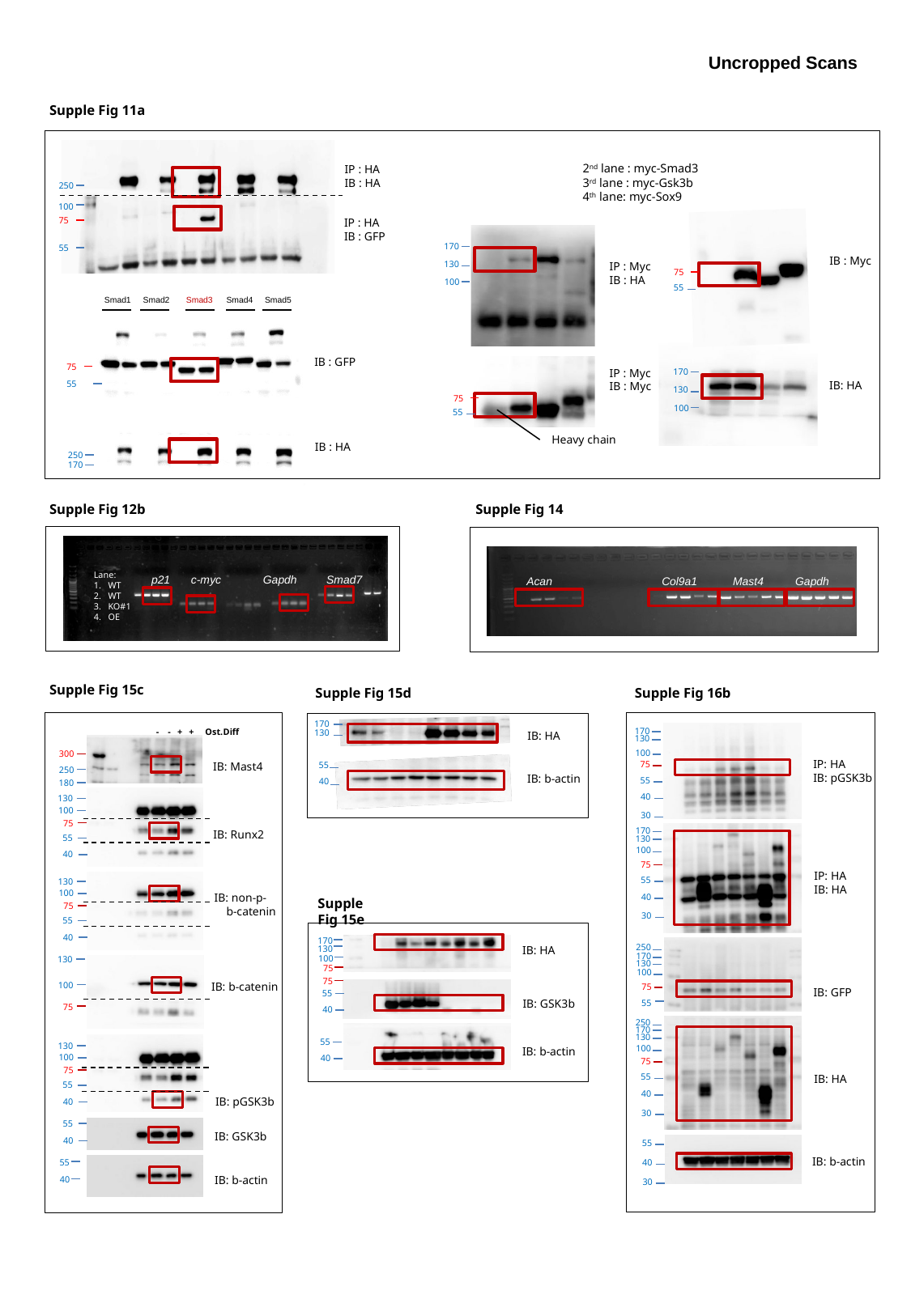

Uncropped Scans
Supple Fig 11a
2nd lane : myc-Smad3
3rd lane : myc-Gsk3b
4th lane: myc-Sox9
IP : HA
IB : HA
250
100
75
IP : HA
IB : GFP
170
55
IB : Myc
130
IP : Myc
IB : HA
75
100
55
Smad1
Smad2
Smad3
Smad4
Smad5
IB : GFP
75
IP : Myc
IB : Myc
170
IB: HA
55
130
75
100
55
Heavy chain
IB : HA
250
170
Supple Fig 12b
Supple Fig 14
Lane:
WT
WT
KO#1
OE
p21
c-myc
Gapdh
Smad7
Acan
Col9a1
Mast4
Gapdh
Supple Fig 15c
Supple Fig 16b
Supple Fig 15d
170
130
IB: HA
55
IB: b-actin
40
170
130
100
IP: HA
IB: pGSK3b
75
55
40
30
170
130
100
75
IP: HA
IB: HA
55
40
30
250
170
130
100
75
IB: GFP
55
250
170
130
100
75
55
IB: HA
40
30
55
IB: b-actin
40
30
- - + + Ost.Diff
300
IB: Mast4
250
180
130
100
75
IB: Runx2
55
40
130
100
IB: non-p-
 b-catenin
75
55
40
130
100
IB: b-catenin
75
130
100
75
55
IB: pGSK3b
40
55
IB: GSK3b
40
55
IB: b-actin
40
Supple Fig 15e
170
130
IB: HA
100
75
75
55
IB: GSK3b
40
55
IB: b-actin
40

## Slide 33
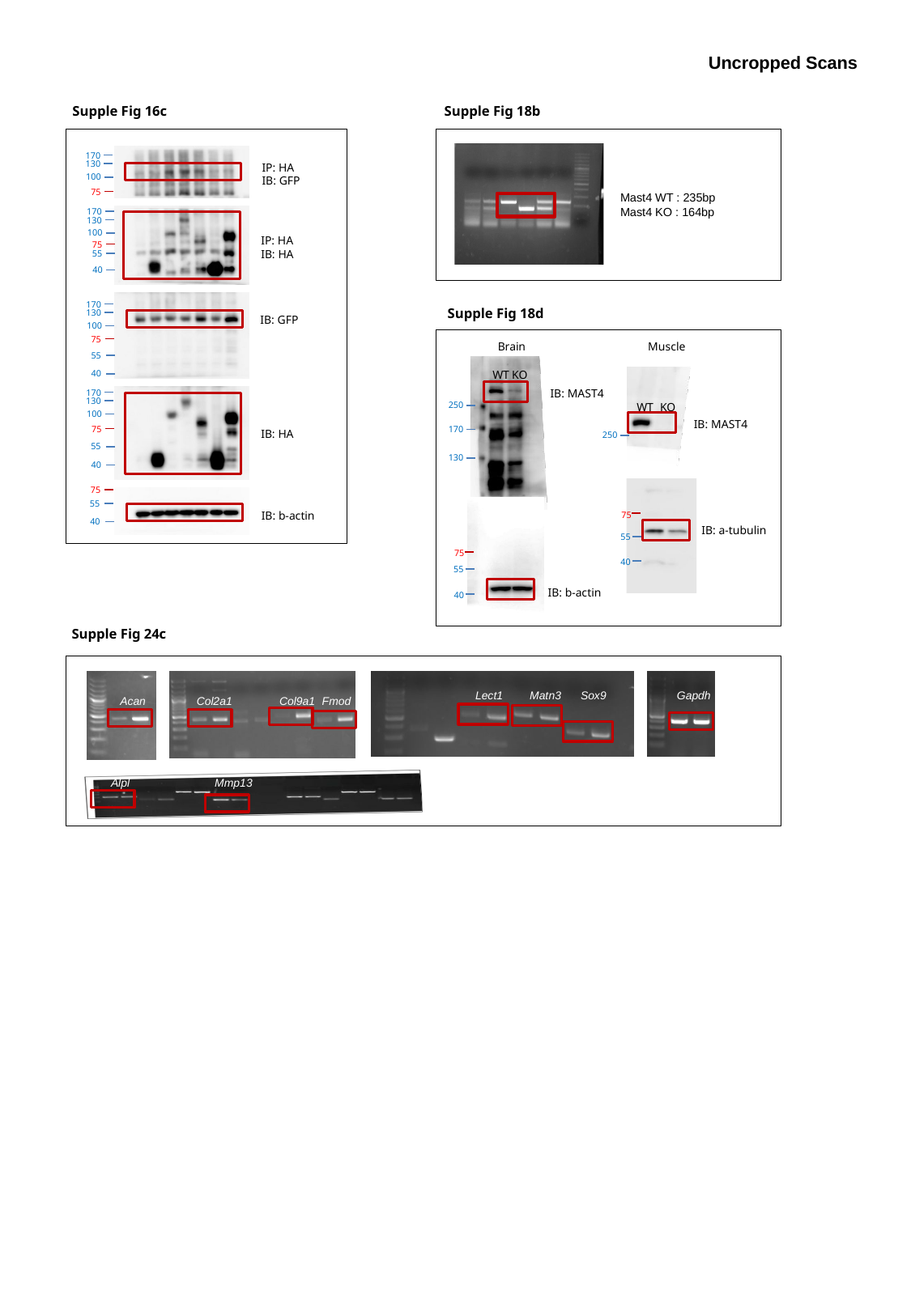

Uncropped Scans
Supple Fig 18b
Supple Fig 16c
170
130
IP: HA
IB: GFP
100
75
170
130
100
IP: HA
IB: HA
75
55
40
170
130
IB: GFP
100
75
55
40
170
130
100
75
IB: HA
55
40
75
55
IB: b-actin
40
Mast4 WT : 235bp
Mast4 KO : 164bp
Supple Fig 18d
Brain
Muscle
WT
KO
IB: MAST4
250
WT
KO
IB: MAST4
170
250
130
75
IB: a-tubulin
55
75
40
55
IB: b-actin
40
Supple Fig 24c
Lect1
Matn3
Sox9
Gapdh
Col2a1
Acan
Col2a1
Col9a1
Fmod
Alpl
Mmp13

## Slide 34
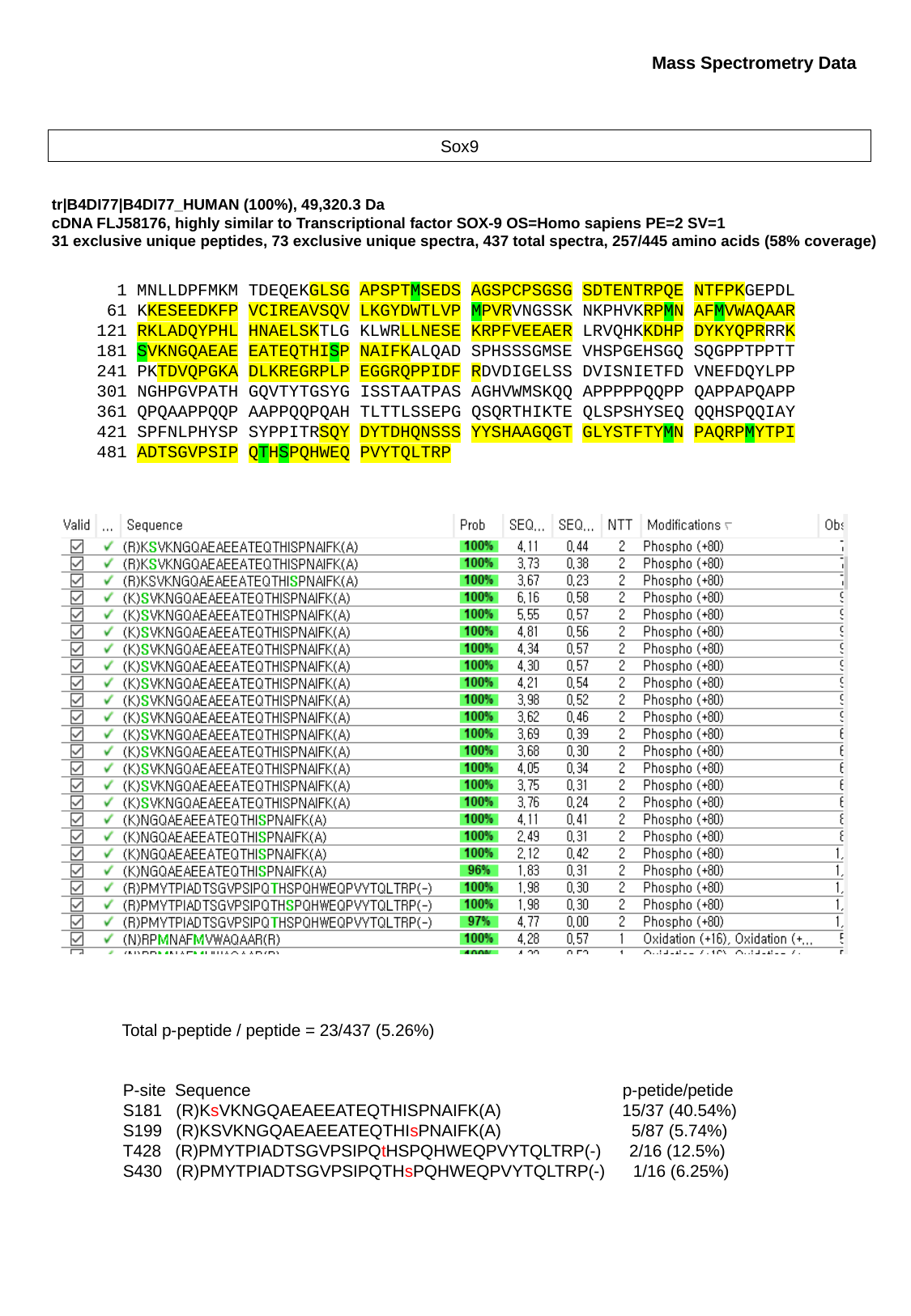

Mass Spectrometry Data
Sox9
tr|B4DI77|B4DI77_HUMAN (100%), 49,320.3 Da
cDNA FLJ58176, highly similar to Transcriptional factor SOX-9 OS=Homo sapiens PE=2 SV=1
31 exclusive unique peptides, 73 exclusive unique spectra, 437 total spectra, 257/445 amino acids (58% coverage)
 1 mnlldpfmkm tdeqekglsg apsptmseds agspcpsgsg sdtentrpqe ntfpkgepdl
 61 kkeseedkfp vcireavsqv lkgydwtlvp mpvrvngssk nkphvkrpmn afmvwaqaar
121 rkladqyphl hnaelsktlg klwrllnese krpfveeaer lrvqhkkdhp dykyqprrrk
181 svkngqaeae eateqthisp naifkalqad sphsssgmse vhspgehsgq sqgpptpptt
241 pktdvqpgka dlkregrplp eggrqppidf rdvdigelss dvisnietfd vnefdqylpp
301 nghpgvpath gqvtytgsyg isstaatpas aghvwmskqq apppppqqpp qappapqapp
361 qpqaappqqp aappqqpqah tlttlssepg qsqrthikte qlspshyseq qqhspqqiay
421 spfnlphysp syppitrsqy dytdhqnsss yyshaagqgt glystftymn paqrpmytpi
481 adtsgvpsip qthspqhweq pvytqltrp
Total p-peptide / peptide = 23/437 (5.26%)
P-site Sequence p-petide/petide
S181 (R)KsVKNGQAEAEEATEQTHISPNAIFK(A) 15/37 (40.54%)
S199 (R)KSVKNGQAEAEEATEQTHIsPNAIFK(A) 5/87 (5.74%)
T428 (R)PMYTPIADTSGVPSIPQtHSPQHWEQPVYTQLTRP(-) 2/16 (12.5%)
S430 (R)PMYTPIADTSGVPSIPQTHsPQHWEQPVYTQLTRP(-) 1/16 (6.25%)

## Slide 35
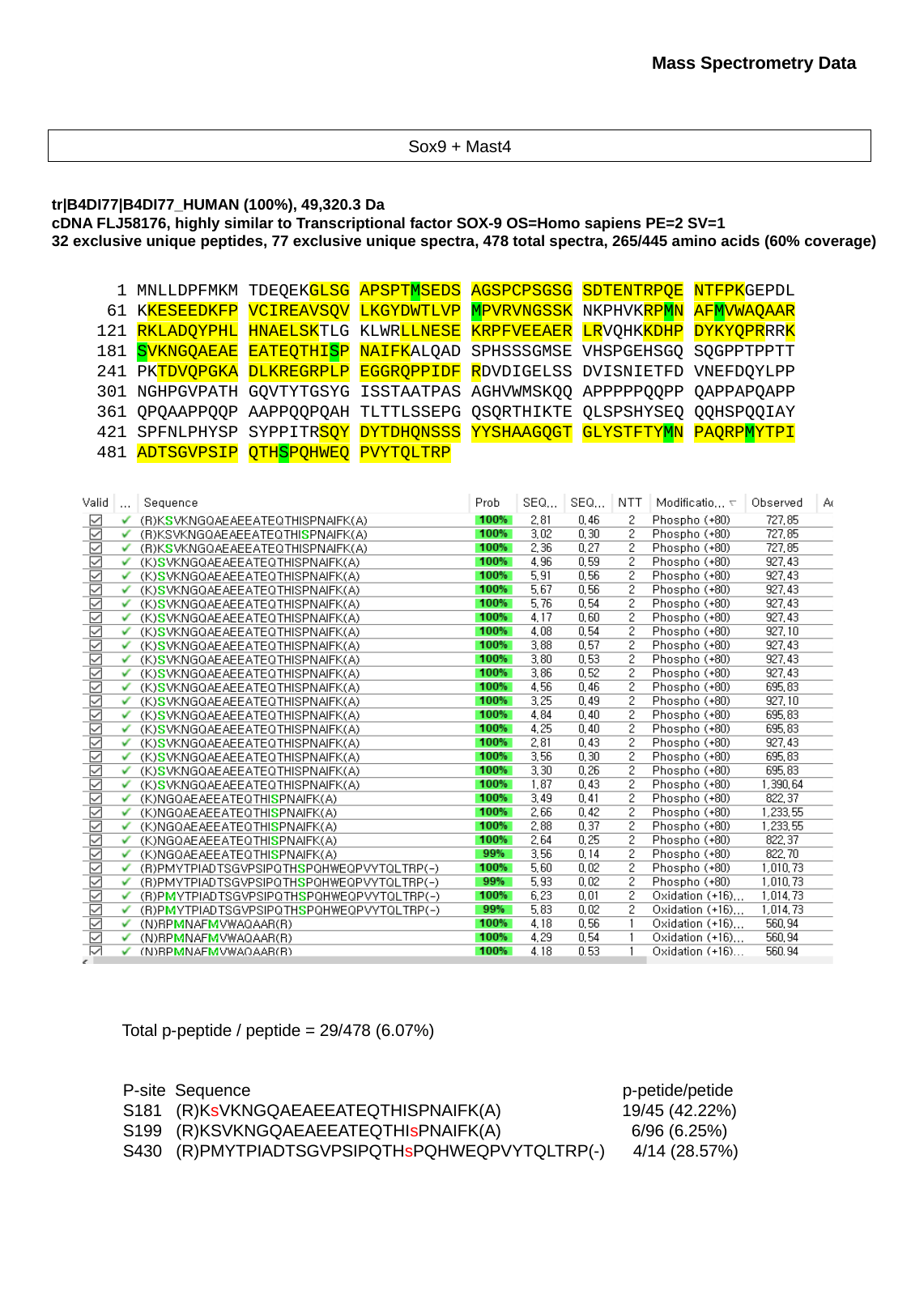

Mass Spectrometry Data
Sox9 + Mast4
tr|B4DI77|B4DI77_HUMAN (100%), 49,320.3 Da
cDNA FLJ58176, highly similar to Transcriptional factor SOX-9 OS=Homo sapiens PE=2 SV=1
32 exclusive unique peptides, 77 exclusive unique spectra, 478 total spectra, 265/445 amino acids (60% coverage)
 1 mnlldpfmkm tdeqekglsg apsptmseds agspcpsgsg sdtentrpqe ntfpkgepdl
 61 kkeseedkfp vcireavsqv lkgydwtlvp mpvrvngssk nkphvkrpmn afmvwaqaar
121 rkladqyphl hnaelsktlg klwrllnese krpfveeaer lrvqhkkdhp dykyqprrrk
181 svkngqaeae eateqthisp naifkalqad sphsssgmse vhspgehsgq sqgpptpptt
241 pktdvqpgka dlkregrplp eggrqppidf rdvdigelss dvisnietfd vnefdqylpp
301 nghpgvpath gqvtytgsyg isstaatpas aghvwmskqq apppppqqpp qappapqapp
361 qpqaappqqp aappqqpqah tlttlssepg qsqrthikte qlspshyseq qqhspqqiay
421 spfnlphysp syppitrsqy dytdhqnsss yyshaagqgt glystftymn paqrpmytpi
481 adtsgvpsip qthspqhweq pvytqltrp
Total p-peptide / peptide = 29/478 (6.07%)
P-site Sequence p-petide/petide
S181 (R)KsVKNGQAEAEEATEQTHISPNAIFK(A) 19/45 (42.22%)
S199 (R)KSVKNGQAEAEEATEQTHIsPNAIFK(A) 6/96 (6.25%)
S430 (R)PMYTPIADTSGVPSIPQTHsPQHWEQPVYTQLTRP(-) 4/14 (28.57%)
