# Supplementary material for: Mast4 determines the cell fate of MSCs for bone and cartilage development
Source: Nat Commun. 2022 Jul 8;13:3960. doi: 10.1038/s41467-022-31697-3 (PMC9270402; doi:10.1038/s41467-022-31697-3)
Supplement: Supplementary file 3 — Reporting Summary [file 41467_2022_31697_MOESM3_ESM.pdf]

nature portfolio

Corresponding author(s): Seong-jin, Kim

Last updated by author(s): Apr 10, 2022

Reporting Summary

Nature Portfolio wishes to improve the reproducibility of the work that we publish. This form provides structure for consistency and transparency in reporting. For further information on Nature Portfolio policies, see our Editorial Policies and the Editorial Policy Checklist.

Statistics

For all statistical analyses, confirm that the following items are present in the figure legend, table legend, main text, or Methods section.

n/a	Confirmed	
☐	☒	The exact sample size (*n*) for each experimental group/condition, given as a discrete number and unit of measurement
☐	☒	A statement on whether measurements were taken from distinct samples or whether the same sample was measured repeatedly
☐	☒	The statistical test(s) used AND whether they are one- or two-sided<br>*Only common tests should be described solely by name; describe more complex techniques in the Methods section.*
☒	☐	A description of all covariates tested
☐	☒	A description of any assumptions or corrections, such as tests of normality and adjustment for multiple comparisons
☐	☒	A full description of the statistical parameters including central tendency (e.g. means) or other basic estimates (e.g. regression coefficient) AND variation (e.g. standard deviation) or associated estimates of uncertainty (e.g. confidence intervals)
☐	☒	For null hypothesis testing, the test statistic (e.g. *F*, *t*, *r*) with confidence intervals, effect sizes, degrees of freedom and *P* value noted<br>*Give P values as exact values whenever suitable.*
☒	☐	For Bayesian analysis, information on the choice of priors and Markov chain Monte Carlo settings
☒	☐	For hierarchical and complex designs, identification of the appropriate level for tests and full reporting of outcomes
☒	☐	Estimates of effect sizes (e.g. Cohen's *d*, Pearson's *r*), indicating how they were calculated

Our web collection on statistics for biologists contains articles on many of the points above.

Software and code

Policy information about availability of computer code

Data collection	Western blotting and Gel Detection : Amersham Imager 600 (GE Healthcare Life Sciences)<br>Real-time PCR : QuantStudio 5 Real-Time PCR Software (Applied Biosystems)<br>Luciferase activity quantification : i-control software, Plate reader Infinite 200 PRO (Tecan)<br>Beta galactosidase activity quantification : Softmax Pro (version 5), VersaMax Microplate reader (Molecular Devices)
Data analysis	GraphPad Prism 5 Software, Image J(64 bit Java 1.80_172) were used for Data analysis. Gene expression was calculated as FPKM using Cufflinks. Differential expression analysis between the wild-type and Mast4-depleted samples was performed by using Cuffdiff. Gene ontology (GO) enrichment analysis for DEG datasets was performed by DAVID. micro CT images were analyzed using Comprehensive TeX Archive Network (CTAN) topographic reconstruction software. FlowJo v10.7.1 and BD FACSDiva v9.0.1 software were used for analysis of Flow cytometry.

For manuscripts utilizing custom algorithms or software that are central to the research but not yet described in published literature, software must be made available to editors and reviewers. We strongly encourage code deposition in a community repository (e.g. GitHub). See the Nature Portfolio guidelines for submitting code & software for further information.

Data

Policy information about availability of data

All manuscripts must include a data availability statement. This statement should provide the following information, where applicable:

- Accession codes, unique identifiers, or web links for publicly available datasets
- A description of any restrictions on data availability
- For clinical datasets or third party data, please ensure that the statement adheres to our policy

The raw data for RNA sequencing generated in this study have been deposited in the NCBI SRA database under accession code SRR12095157 - SRR12095158 for Figs. 1c, 1f, EDF5-6, and SRR12095360 - SRR12095363 for Figs. 6a-c, EDF25-26 (https://trace.ncbi.nlm.nih.gov/Traces/sra/sra.cgi?view=run_browser&run=SRR_number [i.e. SRR number: SRR12095157]). Source data are provided with this paper. The source data for Figs. 1b,d,e, 2c,d, j, 3b,d,e, 4l, 5e,f, 7a,d and EDF 2d, 3b, 7b, 8b, 9a,b, 10, 11b,c, 12a, 13b, 17, 19c,d,e, 20b,d, 23b,c, 26b,d, 28b,c, and 29b have been provided as Source Data file. Unprocessed original scans of blots in Figs. 1a,b, 2b,e,f,g,i,l, 3a,c,f,g, 4b,c,d,f,g,h,i,j,k, 6d,e are shown in Source Data file. Unprocessed original scans of blots in EDF 2b,c,e, 4b, 7e, 9c,d,e, 12b, 14, 15c,d,e, 16b,c, 18d, and 24c and the mass spectrometry data in Fig. 2h are shown at the end of the Supplementary Information file.

Interaction for genes related to cartilage and/or bone development, BMP signaling, TGF-β signaling, and Wnt signaling was searched using STRING database (https://string-db.org/) with high confidence score (≥0.7)

Field-specific reporting

Please select the one below that is the best fit for your research. If you are not sure, read the appropriate sections before making your selection.

☒ Life sciences ☐ Behavioural & social sciences ☐ Ecological, evolutionary & environmental sciences

For a reference copy of the document with all sections, see nature.com/documents/nr-reporting-summary-flat.pdf

Life sciences study design

All studies must disclose on these points even when the disclosure is negative.

Sample size	For in vitro experiments, sample size was three to six replicates, which was determined based on researchers' experience and widely used numbers of experimental replicates in the published literature.<br><br>For animal experiments, a sample size of at least 3 or more was used, and there was a limit to increasing the sample size significantly due to the nature of the use of sex-matched littermates.
Data exclusions	On principle, data were only excluded for failed experiments, reasons for which included suboptimal activation and microbial contamination.
Replication	All experiments were repeated at least three independent experiments, and were replicated by independent researchers. The data shown in the manuscript are representative of at least successful experiments.
Randomization	Most experiments carried out in our study are cell-based molecular work. For in vitro experimets, no randomization was necessary since cells were counted before seeding for differentiation or other experiments (Treatment or transfection, etc), so equal amounts of cells were assured in experimetal or control wells. No randomization of mice. Mice analyzed were littermates and sex-matched.
Blinding	Most of the in vitro experiments using cell (with Knock out or Overexpression) were difficult to apply blinding, but the results were interpreted without bias, and repeated experiments were performed three or more times. The experiments were designed based on preliminary data and pilot experiments.<br><br>The transplantation experiment using rabbit was conducted by a related company, and technicians performed the procedure in complete blindness.

Reporting for specific materials, systems and methods

We require information from authors about some types of materials, experimental systems and methods used in many studies. Here, indicate whether each material, system or method listed is relevant to your study. If you are not sure if a list item applies to your research, read the appropriate section before selecting a response.

Materials & experimental systems

n/a	Involved in the study
☐	☒ Antibodies
☐	☒ Eukaryotic cell lines
☒	☐ Palaeontology and archaeology
☐	☒ Animals and other organisms
☒	☐ Human research participants
☒	☐ Clinical data
☒	☐ Dual use research of concern

Methods

n/a	Involved in the study
☒	☐ ChIP-seq
☐	☒ Flow cytometry
☒	☐ MRI-based neuroimaging

Antibodies

Antibodies used

Supplementary Table 1 provided with manuscript contains information on all antibodies used in the study.

Validation

Antibodies used have been used in the literature and also validated by manufacturers.
alpha-tubulin, Sigma aldrich, B-5-1-2 was validated here : https://www.scbt.com/ko/p/alpha-tubulin-antibody-b-5-1-2
beta-catenin, Cell signaling Technology, D10A8 was validated here : https://www.cellsignal.com/products/primary-antibodies/b-catenin-d10a8-xp-rabbit-mab/8480
beta-actin, Sigma aldrich, AC-15 was validated here : https://www.sigmaaldrich.com/KR/ko/product/sigma/a5441
CD31, BD Pharmingen, 553373 was validated here : https://www.bdbiosciences.com/en-us/products/reagents/flow-cytometry-reagents/research-reagents/single-color-antibodies-ruo/pe-rat-anti-mouse-cd31.553373
Col2a1, Santa cruz biotechnology, M2139 was validated here : https://www.scbt.com/ko/p/col2a1-antibody-m2139
Flag, Roche, M2 was validated here : https://www.sigmaaldrich.com/KR/ko/product/sigma/f3165?gclid=Cj0KCQjwgMqSBhDCARIsAIIVN1UzDmItyvH8ukCko-a2OKWdreEN_IMMvQamJEJni8CG3rsg3ChiuR4aAhVFEALw_wcB
GFP, abcam, ab6556 was validated here : https://www.abcam.com/gfp-antibody-ab6556.html
Gsk-3beta, Cell signaling Technology, 27C10 was validated here : https://www.cellsignal.com/products/primary-antibodies/gsk-3b-27c10-rabbit-mab/9315
GST, Santa cruz biotechnology, B-14 was validated here : https://www.scbt.com/ko/p/gst-antibody-b-14
HA, Santa cruz biotechnology, F-7 was validated here : https://www.scbt.com/ko/p/ha-probe-antibody-f-7
Lamin B, Santa cruz biotechnology, B-10 was validated here : https://www.scbt.com/ko/p/lamin-b1-antibody-b-10
Mast4, Bioworld technology, BS5791 was validated here : https://www.bioworlde.com/pdf/BS5791.pdf
Mast4, Abclon, Customized antibody : Since this antibody was custom-made by us, we directly validated it. This antibody successfully detected the transfected Exogenouse Mast4.
Mmp13, Abcam, ab39012 was validated here : https://www.abcam.com/mmp13-antibody-ab39012.html
Myc, Santa cruz biotechnology, 9E10 was validated here : https://www.scbt.com/ko/p/c-myc-antibody-9e10
non-p-beta-catenin, Cell signaling Technology, D13A1 was validated here : https://www.cellsignal.com/products/primary-antibodies/non-phospho-active-b-catenin-ser33-37-thr41-d13a1-rabbit-mab/8814
Osterix, Abcam, ab22552 was validated here : https://www.abcam.com/sp7--osterix-antibody-ab22552.html
phospho-Gsk-3beta, Cell signaling Technology, 5B3 was validated here : https://www.cellsignal.com/products/primary-antibodies/phospho-gsk-3b-ser9-5b3-rabbit-mab/9323
pSerine, Sigma aldrich, PSR-45 was validated here : https://www.sigmaaldrich.com/KR/ko/product/sigma/p5747
Runx2, Cell signaling Technology, D1L7F was validated here : https://www.cellsignal.com/products/primary-antibodies/runx2-d1l7f-rabbit-mab/12556
Runx2, R&D, MAB2006 was validated here : https://www.rndsystems.com/products/human-runx2-cbfa1-antibody-232902_mab2006
Smad3, Abcam, ab28379 was validated here : https://www.abcam.com/smad3-antibody-ab28379.html
Sox9, Abcam, ab3697 was validated here : https://www.abcam.com/sox9-antibody-ab3697.html
Sox9, Cell signaling Technology, #82630 was validated here : https://www.cellsignal.com/products/primary-antibodies/sox9-d8g8h-rabbit-mab/82630
Sox9, Millipore, AB5535 was validated here : https://www.merckmillipore.com/KR/ko/product/Anti-Sox9-Antibody,MM_NF-AB5535

Eukaryotic cell lines

Policy information about cell lines

Cell line source(s)

human embryonic kidney cell line, HEK293T, murine mesenchymal stem cell line C3H10T1/2 and prosteoblast cell line MC3T3-E1 cells were obtained from the American Type Culture Collection (ATCC). ATDC5, chondrogenic cell line was obtained from RIKEN BRC.

The human primary chondrocytes(hPC) and human bone marrow-derived stem cells (hBMSC) were kindly provided by SCM Lifescience (Incheon, S.Korea), where established hBMSC lines through the subfractionation culturing method (Yi, T. et al. 2015)

Authentication

Cell line authentication was confirmed by short tandem repeat (STR)-profiling

Mycoplasma contamination

All cell lines tested negative for mycoplasma contamination.

Commonly misidentified lines (See ICLAC register)	No commonly misidentified lines were used in this study.

Animals and other organisms

Policy information about studies involving animals; ARRIVE guidelines recommended for reporting animal research

Laboratory animals	Mus musculus, C57BL6/J, Mast4 -/-, PN1, 3 weeks, 5 weeks, 6 weeks old, male, female<br>Mus musculus, Athymic nude mice, 6 weeks old, female<br>Rabbit, New Zealand white rabbits, 5 months old, male<br><br>The laboratory mice were maintained on a 12-h light/dark cycle at room temperature (20-22°C) with constant humidity (40 ± 10%).
Wild animals	The study did not involve wild animals.
Field-collected samples	The study did not involve samples collected from the field
Ethics oversight	All procedures were approved by Institutional Animal Care and Use Committee of KNOTUS Co., Ltd, Institutional Animal Care and Use Committee of Center for Phenogenomics Animal Research Facility and Woojung BSC (ssociation for Assessment and Accreditation of Laboratory Animal Care-accredited facility).

Note that full information on the approval of the study protocol must also be provided in the manuscript.

Flow Cytometry

Plots

Confirm that:

- [x] The axis labels state the marker and fluorochrome used (e.g. CD4-FITC).
- [x] The axis scales are clearly visible. Include numbers along axes only for bottom left plot of group (a 'group' is an analysis of identical markers).
- [x] All plots are contour plots with outliers or pseudocolor plots.
- [x] A numerical value for number of cells or percentage (with statistics) is provided.

Methodology

Sample preparation	Mast4+/+ and Mast4-/- mice (n=5) at 5 weeks of age were sacrificed, followed by dissection of humerus, femur and tibia. Cells were isolated with a combination of mechanical and chemical digestion, and red blood cells were removed by ammonium–chloride–potassium (ACK) lysis buffer. The TER119+CD45+ hematopoietic cells were filtered by magnetic-activated cell sorting (MACS) before FACS sorting.
Instrument	FACS analysis was performed on an FACS Aria II Instrument (BD Biosciences)
Software	FlowJo v10.7.1 and BD FACSDiva v9.0.1 software were used for analysis
Cell population abundance	Post-sort analysis using same gating strategy was performed to determine the purity of the sorted cell populations. Sorted cells were 74.5% in Mast4+/+ and 73.0 % in Mast4-/- mice when FACS analysis was completed.
Gating strategy	i) SSC-A versus FSC-A to capture all cells; (ii) FSC-W versus FSC-H to capture singlets; (iii) SSC-W versus SSC-H to capture singlets; (iv) SSC-A versus CD45/TER119/7-AAD to capture all live (7-AAD−), non-hematopoietic (CD45−), non-red blood cells (Ter119−) (v) TIE2 versus ITGAV to capture the TIE2−ITGAV + population; (vi) THY1 versus 6C3 to capture the THY1−6C3− population and (vii) CD105 versus to ITGAV to capture the CD105-ITGAV population (p-mSSC).

- [x] Tick this box to confirm that a figure exemplifying the gating strategy is provided in the Supplementary Information.
